# Mapping and cloning of pepper fruit color-related genes based on BSA-seq technology

**DOI:** 10.3389/fpls.2024.1447805

**Published:** 2024-10-25

**Authors:** Shuo Feng, Ling Zhou, Rahat Sharif, Weiping Diao, Jiali Liu, Xinxin Liu, Kunhao Chen, Guoju Chen, Bihao Cao, Zhangsheng Zhu, Yi Liao, Jianjun Lei, Changming Chen

**Affiliations:** ^1^ Key Laboratory of Biology and Genetic Improvement of Horticultural Crops (South China), Ministry of Agriculture and Rural Affairs, College of Horticulture, South China Agricultural University, Guangzhou, China; ^2^ Jiangsu Key Laboratory for Horticultural Crop Genetic Improvement, Institute of Vegetable Crops, Jiangsu Academy of Agricultural Sciences, Nanjing, Jiangsu, China; ^3^ Guangdong Helinong Biological Seed Industry Co., Ltd, Shantou, Guangdong, China

**Keywords:** pepper, fruit color, BSA-seq, genetic mapping, *CapCCS*

## Abstract

Fruit color is an important qualitative trait that greatly influences the marketability of peppers. Fruit color can be divided into two categories. Green fruit color denotes commercial maturity, whereas ripe fruit indicates physiological maturity. Herein, segregation populations were created using the ‘D24’ with pale green in the green fruit stage, orange in the mature fruit stage, and ‘D47’ with green in the green fruit stage and red in the mature fruit stage. BSA-seq and genetic linkage map analysis revealed green fruit color was linked to (*gyqtl1.1*) on Chr1 and (*gyqtl10.1*) on Chr10, while mature fruit color was linked to Chr6. Using functional annotation, sequence, and expression analysis, we speculate that an SNP mutation in the *CapGLK2* gene at the *gyqtl10.1* interval could initiate premature termination of translation, thus yielding green to pale green fruits in D47. Conversely, the orange color in mature D24 fruits is due to the Indel-mediated premature termination of translation of the *CapCCS* gene. Our research offers a theoretical foundation for choosing different varieties of pepper fruit based on their color.

## 1 Introduction

Peppers (*Capsicum* spp.), a member of the Solanaceae family, are extensively grown in many parts of the world. They can be either annual or limited perennial plants and are frequently cross-pollinated. There are five important cultivated species of pepper, namely *C. annuum*, *C. frutescens*, *C. chinense*, *C. baccatum*, and *C. pubescens*. Pepper fruit color is a key agronomic and economic trait, directly attracting the attention of consumers and breeders (Liu et al., 2022). Pepper fruits can be classified into two groups based on their developmental stages: green fruits, which reach the commercial maturity stage (25-40 DAF), and mature fruits, which get to the physiological maturity stage (40-65 DAF) (Wahyuni et al., 2011). The diverse pigments are responsible for the coloration of pepper fruits and are associated with peppers’ quality, nutritional content, health benefits, and flavor profile. These pigments have extensive use in food, medicine, and chemical industries (Alvarez-Parrilla et al., 2011; Wahyuni et al., 2013).

Pepper at the green fruit stage displays a wide range of colors. Chlorophyll is the primary pigment, whereas trace amounts of carotenoids, flavonoids, and other pigments are also determined at the green fruit stage (Liu et al., 2020). Previous research found that two quantitative trait loci (QTL), *pc1* and *pc10*, respectively positioned on chromosomes 1 and 10, affect the color of premature fruit. The loss of function of the *ARABIDOPSIS PSEUDO RESPONSE REGULATOR2-LIKE* (*APRR2-like*) gene causes a drop in chlorophyll concentration, which in turn prompts the pepper fruits to turn white during the green fruit stage (Brand et al., 2012; Borovsky et al., 2019; Pan et al., 2013). BSA-seq revealed the *LSD ONE LIKE1* (*LOL1*) gene as a key candidate for *pc1*, controlling chloroplast size and chlorophyll concentration (Borovsky et al., 2019). The *GOLDEN LIKE 2* (*GLK2*), a prominent candidate gene for *pc10.1*, regulates chloroplast size, affecting the chlorophyll concentration of pepper peels (Brand et al., 2014). The *CaPP2C35* (*Capana10g00171*0) gene modulates exocarp chlorophyll accumulation to produce light-green ripe pepper fruits (Wu et al., 2022). Genetic linkage studies pinpointed the *ly* locus on Chr9 that controls the color of immature pepper fruit. The decreased carotenoid content in immature pepper fruits could be due to a nonsense mutation in the candidate gene of *ly* locus, *APRR2* (Song et al., 2022).

The process of chloroplasts converting into chromoplasts during ripening is responsible for the color of ripe fruits. Carotenoids are synthesized and stored in chromoplasts. The accumulation of carotenoids leads to the development of orange, red, and yellow colors in ripe pepper fruits (Martí et al., 2009). Three loci, *C1*, *C2*, and *Y*, primarily regulate the color of peppers’ mature fruits (Hurtado-Hernandez and Smith, 1985; Kormos, 1960). Mutations or deletions in the *CCS* gene, co-segregated with the *Y* locus, cause yellow-green fruits that cannot produce capsorubin (a major biomolecule responsible for red pigmentation) (Popovsky and Paran, 2000; Lefebvre et al., 1998). Molecular markers using a segregating red and orange pepper population revealed that the *PSY* gene and the F_2_ phenotype co-segregated (Huh et al., 2001). The *PRR2* gene was identified in a segregating yellow and white mature pepper fruit population. At the *C1* locus, a nonsense mutation in the *PRR2* gene was found to be linked to the color of the fruit (Jeong et al., 2020). Aside from the primary three loci, several genes can regulate the color of mature pepper fruit. For example, the *PSY2* may compensate for the loss of *PSY1*, resulting in a yellow pepper fruit phenotype (Jang et al., 2020).

Recently, genome sequencing of numerous cultivated pepper cultivars has been performed as sequencing technology has improved and costs have decreased. It establishes a basis for researching genetic mapping, gene localization, cloning, and the production of molecular markers (Qin et al., 2014; Liao et al., 2022). The BSA-seq, in conjunction with molecular markers, has been extensively employed for genetic analysis and gene mapping in peppers. In this regard, an F_2_ segregating population of mature peppers with dark and light green fruit colors was generated (Song et al., 2022). Through BSA-seq and genetic mapping, they successfully mapped a novel interval on Chr9 and predicted four candidate genes.

This study constructed a genetic population by crossing two pepper varieties. One variety, D24 (female parent), has a pale green color at the green stage and an orange color at maturity. The other, D47 (male parent), has a green color at the green fruit stage and a red color at the mature stage. The physical and genetic intervals associated with pepper color traits, such as green and mature fruit colors, were mapped using BSA-seq. We then refined the mapping intervals and identified candidate genes regulating pepper fruit color. The findings of this study provide valuable references and a theoretical basis for breeding pepper varieties with desirable fruit colors.

## 2 Materials and methods

### 2.1 Plant materials

This study utilized high-inbred pepper cultivars D24 (*C. annuum*) and D47 (*C. annuum*) as plant materials. The green fruit of D24 peppers is pale green, and the mature fruit color is orange. On the other hand, the immature fruit of D47 peppers displayed a green color, whereas the mature fruit exhibited a red color. The F_1_ generation was obtained by crossing D24 as the maternal parent (P_1_) with D47 as the paternal parent (P_2_). Self-pollination of the F_1_ plants resulted in forming the F_2_ segregating population. Professor Weiping Diao’s research group at the Vegetable Research Institute of Jiangsu Academy of Agricultural Sciences provided all the plant materials used in this research. The plants were cultivated inside greenhouses at the Vegetable Base of South China Agricultural University (**Supplementary Figure 1**).

### 2.2 Phenotypic identification and genetic analysis

For each plant of P_1_, P_2_, F_1_, and F_2_, three or more green and ripe fruits were selected to determine the color of fruit skin using both visual assessment and spectrophotometric analysis. Visual assessment was conducted by human observation, while spectrophotometric analysis employed an automated color difference meter (CHROMA METER CR-400). Three equidistant points were measured along the equatorial surface of each fruit to obtain relevant color difference indices. The CIE Lab (Commission Internationale de I’Eclairage Lab* color space values) color system was utilized to quantify the color indices of the pepper fruit surface. The average value of each index was calculated as the measured value for subsequent analysis. Statistical analysis of the obtained results was performed using Microsoft Excel 2016.

### 2.3 Extraction and determination of pigments

We took three fruits from each plant that showed varying fruit colors in the F_2_ population. Pigments were extracted using the direct immersion method for plant tissues (Wellburn and Lichtenthaler, 1984). The absorbance values of the extraction solution were measured using a Cytation5 microplate reader. Chlorophyll and carotenoids were measured using the absorbance values. Three biological replicates were performed for each sample. The CORREL function in Microsoft Excel 2016 was employed to calculate the correlation coefficients between the color difference parameters (*h*, *C*, *L*, and *E* values) of the green and mature fruits in the F_2_ population and their corresponding chlorophyll and carotenoid contents. The T statistic was calculated using the SQRT function based on its formula. Finally, the T.INV function was used to determine the critical values of T at the 99% and 95% confidence levels. The significance level was determined by comparing the calculated T statistic with the critical T values.

### 2.4 BSA analysis of pepper fruit color

The D24 and D47 pepper varieties were chosen as parental pools in this experiment. After investigating and performing statistical analysis on the fruit color traits of the F_2_ population, two extreme pools were created. Each pool consisted of 30 plants displaying green colors, specifically green and pale green peppers, and another 30 plants exhibiting mature colors, including red and orange peppers. One young leaf of equal size was collected from each selected plant.

DNA extraction from the two parents and pools was done using the cetyl-trimethyl-ammonium-bromide (CTAB) method (Allen et al., 2006). Paired-end sequencing libraries with insert sizes of 350 bp were prepared for sequencing on the MGI-SEQ 2000 sequencing platform (MGI Tech Co., Ltd., Shenzhen, China) by Frasergen Biotechnology Co., Ltd. (Wuhan, China), resulting in pair-end sequence data (2×150 bp). Subsequently, the raw reads were subjected to cleaning using SOAPnuke (Chen et al., 2018), and the cleaned reads were aligned to the “CA59” pepper genome using BWA-mem (version 0.7.17) (Li and Durbin, 2009) and SAMtools (version 1.13) (Li et al., 2009). PCR duplicate reads were marked and filtered using the MarkDuplicates function of Picard v2.25.7 software (http://broadinstitute.github.io/picard). SNP calling and InDel calling were performed using BCFtools (version 1.12) (Li et al., 2009), followed by filtering with the parameter -e ‘%QUAL<10 || (RPBZ<0.1 && %QUAL<15) || (AC<2 && %QUAL<15) || MQ < 30 || MQSBZ <=0.1’. Variants were annotated using SnpEff (version 5.0) software (Cingolani et al., 2012). The SNP-index values of the two pools were obtained using the QTLseqr package (Mansfeld and Grumet, 2018) based on the method proposed by Takagi (Takagi et al., 2013). The Δ(SNP-index) was calculated by subtracting the SNP-index of the two pools. The average value of Δ(SNP-index) for each 4 Mb window with a 10 kb step size was determined. Thresholds of 95% and 99% confidence levels were chosen to identify potential QTL candidates by examining the distribution of Δ(SNP-index) on the chromosomes of the two pools. Regions above the confidence line with Δ(SNP-index) > 95% were marked as potential QTL candidates.

### 2.5 Screening of polymorphic InDel Markers and construction of genetic linkage map

InDel markers within the targeted regions obtained from BSA-seq were selected, and specific primers were designed using Primer Premier 5 software. The synthesis of primers was assigned to Beijing Qingsci Biotechnology Co., Ltd. (**Supplementary Tables 1**–**4**). Subsequently, PCR amplification was conducted on the InDel markers using D24 (P_1_), D47 (P_2_), and F_1_ plants as templates. Polymorphic InDel markers were identified through gel electrophoresis using a 3% agarose gel at 160 V voltage in the electrophoresis buffer. Gel bands were observed using a gel imaging system.

Polymorphic InDel markers were further identified, and their band distributions were analyzed in 259 F_2_ individuals. Combining the phenotypic data of the F_2_ population, a genetic linkage map controlling pepper fruit color was constructed using IciMapping software, with an LOD threshold of 5.0 to determine the presence of QTLs.

### 2.6 Functional annotation of candidate genes

Functional annotation of candidate genes was performed using the CDS sequences. The Diamond v2.0.9 (Buchfink et al., 2021) and BLAST v2.11 (Altschul et al., 1990) software aligned the CDS sequences against the NT, NR, and Swissprot databases. The gene descriptions from the alignment results were considered as the annotation information for the genes.

### 2.7 Cloning and expression analysis of *CapCCS* gene

The gene sequence of *CapCCS* and its flanking 200 bp sequences were extracted based on the “CA59” pepper reference genome (Liao et al., 2022). Specific primers were designed using Primer Premier 5 software (**Supplementary Table 5**), and the *CapCCS* gene was cloned with genomic DNA from D24 and D47 plants as templates.

Pepper peel samples were collected during the premature stage, commercial maturity, breaker, and physiological maturity stages of D24 and D47 plants. Three fruits were collected at each stage, and total RNA was extracted using the total RNA extraction kit from Shanghai Promega Biotech Co., Ltd. cDNA was synthesized using the HiScript^®^II Q RT SuperMix for qPCR (+gDNA wiper) reverse transcription kit from Jiangsu Novogene Bioinformatics Technology Co., Ltd. The cDNA was diluted to a concentration of 200 ng/μL, and specific primers for the *CapCCS* gene (**Supplementary Table 6**) were designed. The ubiquitin extension protein gene *CA12g20490* was used as the internal reference gene, and fluorescence quantitative PCR was performed using the ChamQ Universal SYBR qPCR Master Mix from Jiangsu Novogene Bioinformatics Technology Co., Ltd.

The genes in the candidate interval were quantitatively analyzed using the transcriptome data of the existing D24 and D47 peels of the research group, and three biological replicates (unpublished) were taken from the pericarp of each parent. Heatmaps were generated using the TBtools software (Chen et al., 2020).

### 2.8 Cloning analysis of *CapGLK2* gene

The gene sequence of *CapGLK2* and its flanking 200 bp sequences were extracted based on the “CA59” pepper reference genome (Liao et al., 2022). Samples of pepper peel were taken from D24 and D47 plants at commercial maturity. Total RNA was isolated from three fruits collected at each stage using a kit from Shanghai Promega Biotech Co., Ltd. cDNA was synthesized using a HiScript^®^II Q RT SuperMix for qPCR (+gDNA wiper) reverse transcription kit from Jiangsu Novogene Bioinformatics Technology Co., Ltd. The cDNA was diluted to a concentration of 200 ng/μL to enhance the accuracy of SNP verification in the gene sequence. The CapGLK2-AD vector was created by the technique of homologous recombination, and the primers were produced utilizing the cloning web page (https://crm.vazyme.com/cetool/simple.html) provided by Nanjing Novozan Biotechnology Co., Ltd. (**Supplementary Table 5**). The cDNA obtained through reverse transcription was utilized as a template for PCR amplification. The resulting amplification product was then retrieved and ligated with the enzymatically digested AD vector. The resulting linkage product was transformed into *Escherichia coli* through heat shock DH5α culture. Subsequently, colony PCR and sequencing identification were performed to complete the process.

### 2.9 Statistical analysis

The statistical analyses were conducted using ANOVA in the IBM SPSS Statistics program for Windows, version 21.0 (IBM Corporation, Armonk, NY, USA). The data are presented in the form of the mean ± SD. Multiple mean differences are evaluated using Duncan’s multiple range tests with 5% probability. GraphPad Prism 8.0 (GraphPad Software, Inc., LA Jolla, CA, USA) was used for plotting.

## 3 Results

### 3.1 Genetic analysis of green fruit color in peppers

**Figure 1:**
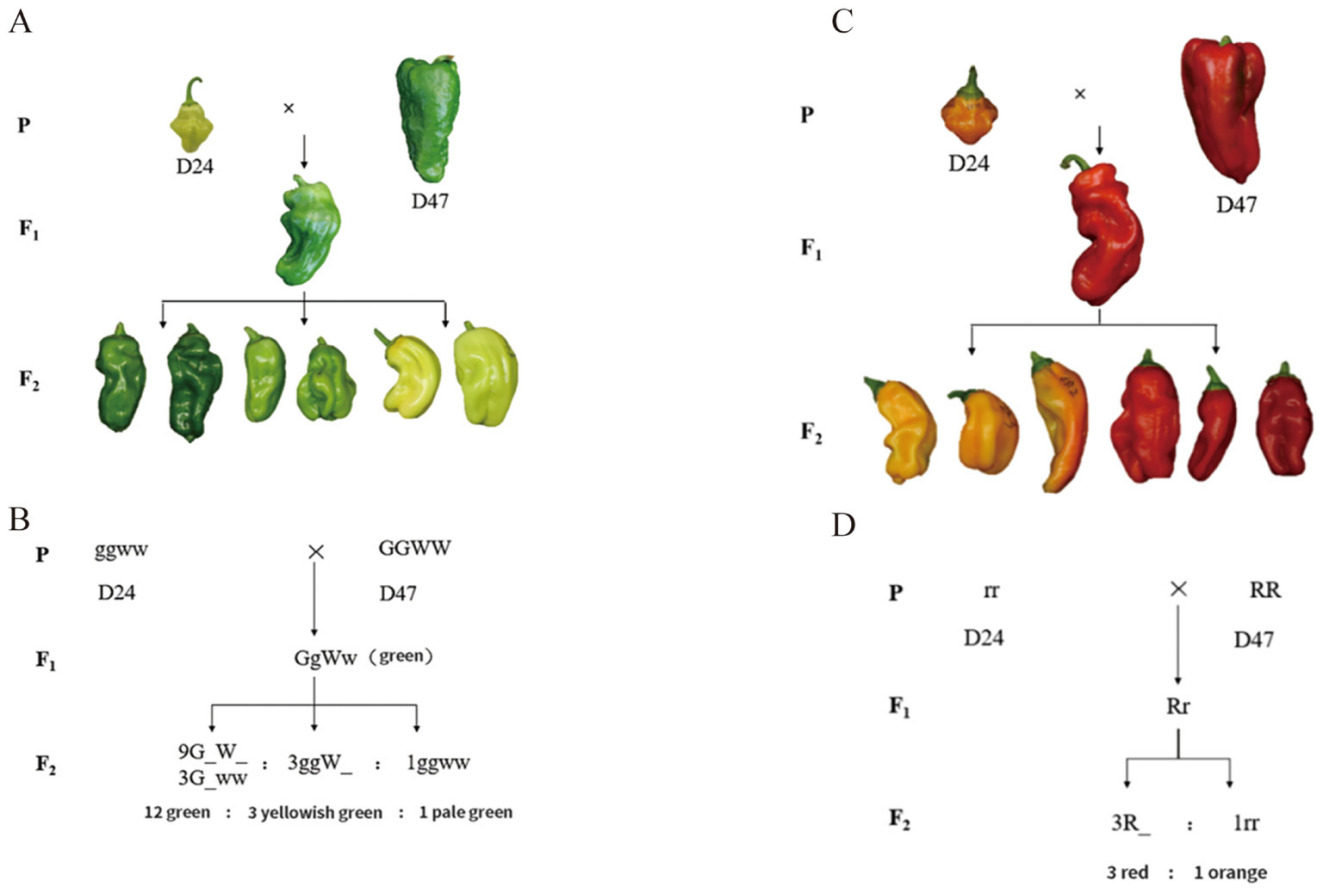
Genetic pattern of the D24 × D47 combination of green and mature fruit color hybrids. **(A)** The genetic pattern of the green fruit color of the D24 × D47 hybrid combination. **(B)** The schematic diagram of the green fruit color of the hybrid combination. **(C)** The genetic pattern of the mature fruit color of the D24 × D47 hybrid combination. **(D)** The schematic diagram of the mature fruit color of the hybrid combination.

According to field investigations, the predominant fruit color in pepper peels is green to pale green. This is supported by the fact that the progeny of a cross between the pale green D24 (*C. annuum*, P_1_) and the green D47 (*C. annuum*, P_2_) are uniformly green in color (**Figures 1A, B**). The F_2_ population exhibited a spectrum of three distinct peel colors: pale green, greenish green, and yellowish green. The segregation pattern in 517 F_2_ individuals was analyzed using chi-square testing, which revealed a ratio of 12:3:1 for green to yellowish green to pale green fruit color. The P-value obtained was 0.38 > 0.05. This suggests the presence of dominant epistasis between two pairs of alleles that govern this trait (**Figures 1C, D**). These findings indicate that two sets of alleles control the color of pepper fruit with interacting effects. The dominant allele for green color supersedes the yellowish-green allele, and the yellowish-green allele supersedes the light green allele.

**Figure 2:**
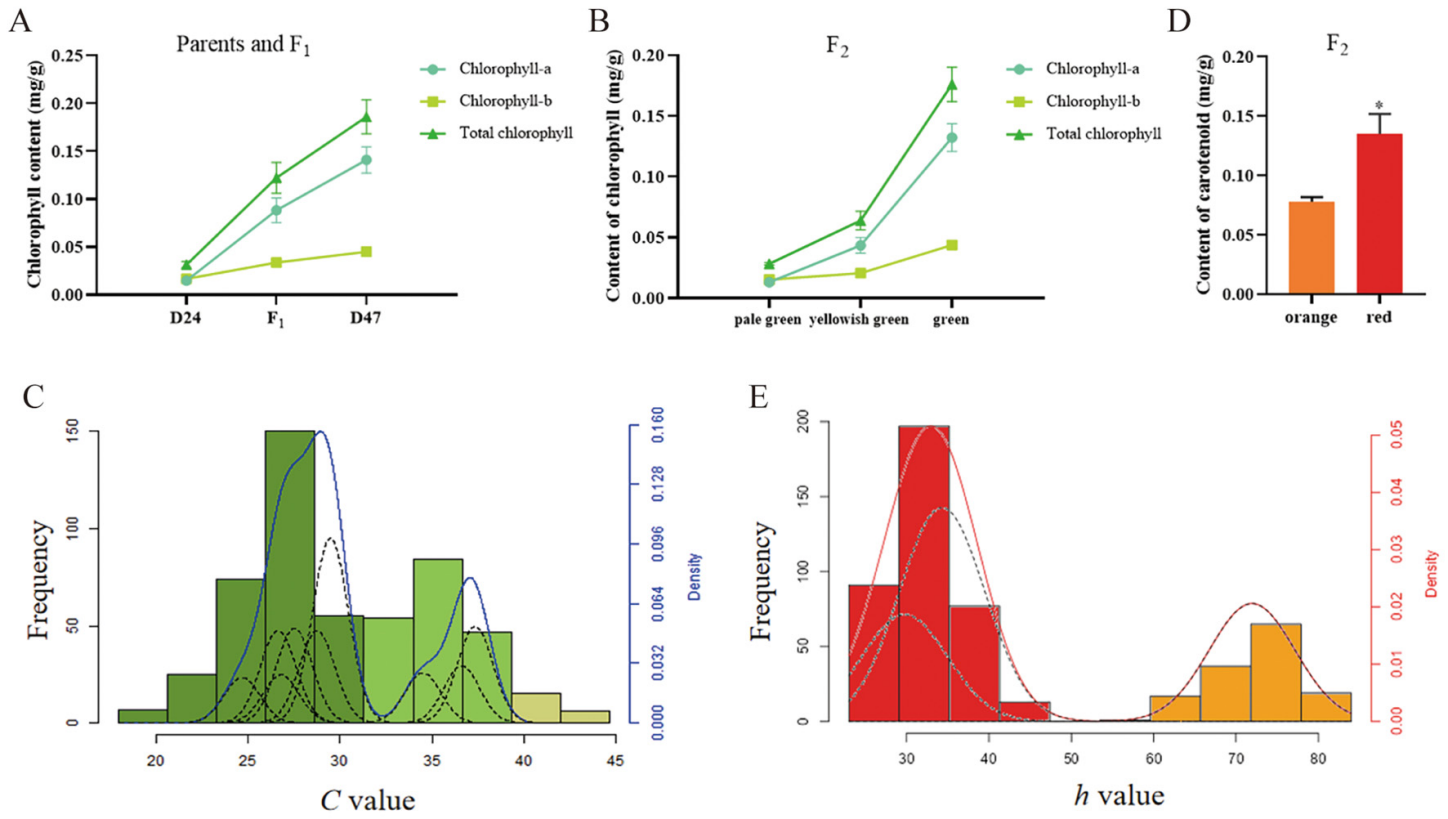
Main pigment content and correlation index of color difference between green and mature fruits of F_2_ generation. **(A)** Parenting D24 and D47 and its hybrid F_1_ pepper in green fruit stage, chlorophyll content in the peel folding drawing. **(B)** The F_2_ Folding diagram of chlorophyll content in various graded fruits in the pepper. **(C)** A frequency distribution plot of *C* values of F_2_ with green fruit. The green columns in the figure represent the green fruit distribution; the light green columns represent the yellowish green fruit distribution; and the yellow plants represent the pale green distribution. The blue solid line is the fitted curve, and the black dashed line is the component distribution curve. **(D)** The statistical plot of carotenoid content in the colored pericarp of F_2_ peppers with mature fruit, Where * indicates significant differences at the 0.05 significance level. **(E)** A frequency distribution plot of h values of F_2_ with mature fruit. The red solid line is the fitted curve, and the black dashed line is the distribution curve of the components.

The chlorophyll content of fruits from P_1_, P_2_, F_1_, and F_2_ plants in each fruit color category was measured using direct immersion. In line with the phenotypic variations between the two parents, the average total chlorophyll content in P_2_ was 0.186 mg/g, about six times more than in P_1_ (0.032 mg/g) (**Figure 2A**). In the F_2_ population, green fruits had an average total chlorophyll content of 0.176 mg/g, yellowish green fruits 0.064 mg/g, and pale green fruits 0.009 mg/g. These values differentiated between the three categories of fruit color (**Figure 2B**), suggesting that visual grading could be used to classify the color of green fruit peel in peppers. The color difference values of green fruit from P_1_ (D24), P_2_ (D47), and their F_1_ and F_2_ generations were measured using a colorimeter. The four indicators of *L*, *C*, *h*, and *ΔE* were also evaluated. The color difference values of P_1_ (D24), P_2_ (D47), and their F_1_ and F_2_ generations were determined by colorimeter to analyze the four indexes of *L*, *C*, *h*, and *ΔE*. According to the data, the *C* value of F_1_ generation tilted toward the P_1_ (D24), while the *L*, *h*, and *ΔE* values showed relevance to P_2_ (D47) (**Supplementary Table 7**). Consistent with quantitative trait features, the results allow for the possibility of using measured values of the color difference index to conduct genetic studies of green and ripe pepper fruit coloration.

We calculated the correlation between the total chlorophyll content of F_2_-graded fruit color and *L, C, h*, and *ΔE*. The correlation coefficient between the *C* value and green fruit peel is the highest, followed by *ΔE*, *L*, and *h* values (**Supplementary Table 8**). The *C* value was used to draw the frequency distribution histogram of the color difference index related to the green fruit color of the F_2_ generation. A bimodal continuous distribution was shown in the histogram (**Figure 2C**). According to the typical genetic characteristics of plant quantitative traits, the green fruit color can be classified into two categories based on the valley between the two peaks. The large peak in this category represents the green peel, while the small peak represents the yellowish-green and pale-green peel. To further evaluate the *C* value of the green mature fruit of the D24 × D47 cross, the major gene and polygene F_2_ single-generation segregation model was utilized. According to the principle of minimizing the AIC value, SEA-F_2_ software was used to select the best model. The best model is 2MG-AD, a two-pair additive-dominant major gene model.

### 3.2 Genetic analysis of mature fruit color in peppers

By crossing the orange-ripe fruit of D24 with the red-ripe fruit of D47, the F_1_ progeny were uniformly red when they reached maturity. This indicates that the red fruit color dominates over the orange fruit color in pepper. Red to orange fruit color was found to be segregated in a 3:1 manner in 517 F_2_ individuals, with a P-value of 0.27 > 0.05. This suggests that a single pair of alleles controls the fruit color trait at the mature stage, with red being completely dominant over orange (**Figures 1C, D**). The carotenoid content in the pepper peel from the F_2_ individuals in each color group at the mature fruit color stage was determined using the direct immersion method. The results indicated that the average carotenoid content in orange and red fruits was 0.080 mg/g and 0.135 mg/g, respectively, with the red fruits exhibiting carotenoid levels twice as high as the orange fruits (**Figure 2D**). The disparity in carotenoid content effectively differentiated the two-color categories, confirming the practicality of employing visual grading for classifying pepper peel color during the ripening stage. A colorimeter was used to measure the *L*, *C*, *h*, and *ΔE* values of mature fruit color of P_1_ (D24), P_2_ (D47), and their F_1_ and F_2_ generations. After Duncun’s multiple comparisons, it was found that there were significant differences in the *L*, *C*, *h*, and *ΔE* values of the two parents. The measured values of *L*, *C*, *h*, and *ΔE* of the four indicators of mature fruit color in the F_1_ generation were significantly different. The average values are also significantly different between the two parents, and they are all biased toward the male parent of the red peel D47 (**Supplementary Table 9**), which is consistent with the characteristics of quantitative traits. Measuring the values of color difference indicators allows for the genetic study of mature pepper fruit color. Calculating the correlation between the total carotenoid content of F_2_-graded mature fruit color and *L, C*, *h*, and *ΔE* shows that the *h* value has the highest correlation coefficient with the peel of mature pepper fruits, followed by *ΔE*, *L*, and *C* value (**Supplementary Table 10**). The *h* value was utilized to construct the frequency distribution histogram of mature fruit color in the F_2_ generation. The histogram (**Figure 2E**) shows a bimodal continuous distribution. The ripe fruit color can be divided into two categories based on the peaks between the valleys. Among these, the orange peel is represented by the small peaks, and the big peaks represent the red peel. The separation between fruit colors is basically in line with the ratio of red: orange = 3:1, indicating that there may be the separation of a pair of major genes, and red versus orange is completely dominant, which is consistent with the results of Mendelian separation analysis.

### 3.3 Linkage analysis between green and mature fruit color

The genetic investigation of pepper peel color revealed that the green, yellowish, green, and pale green fruit color ratio is 12:3:1, respectively. The ratio of red and orange-yellow fruit color is 3:1 in the mature pepper fruit. The linkage between green and mature peppers was analyzed using the chi-square test to further understand the relationship between green and mature fruit color. There was a 3:1 pattern for the red-to-orange ripe fruit color ratio among the 375 plants that displayed green fruit color. Similarly, a 3:1 pattern was seen in red to orange ripe fruit color among the 109 plants with green fruits showing yellowish-green coloration. A 3:1 trend was seen in the red-to-orange ripe fruit color among the 33 green fruit plants, all with pale green coloration. Likewise, the fruit color at the green fruit color stage of the 377 plants bearing red-mature fruits follows the pattern of green: yellowish green: pale green = 12:3:1. Among the 140 plants with orange-mature fruit, the color of the fruit at green fruit color stage with the ratio of green: yellowish green: pale green = 12:3:1 was respectively observed (**Supplementary Table 11**). These results suggest that in the D24 × D47 cross, green and ripe fruit colors are inherited independently.

### 3.4 Preliminary mapping of genes associated with pepper fruit color

This investigation utilized whole-genome resequencing to uncover genetic loci responsible for the difference in fruit color between green and mature peppers. The parental lines, D24 and D47, were subjected to resequencing, along with four pooled populations of F_2_ individuals exhibiting premature (green and yellow) and mature (red and orange) fruit colors. The high-quality reads from D24, D47, green-immature (green), green-immature (yellow), red-mature, and orange-mature fruit pools were effectively aligned to the “CA59” reference genome, with mapping reads of 712,847,132, 722,405,104, 1,073,269,721, 1,133,524,293, 1,042,405,332, and 1,040,385,125, respectively. The resulting average sequencing depths were 34.17×, 34.63×, 51.45×, 54.34×, 49.97×, and 49.87×, correspondingly (**Supplementary Table 12**). These outcomes affirm the satisfactory quality of the sequencing data obtained from all samples, demonstrating a robust alignment efficiency to the “CA59” reference genome. This facilitates the subsequent identification of genetic variations and gene mapping for trait analysis.

**Figure 3:**
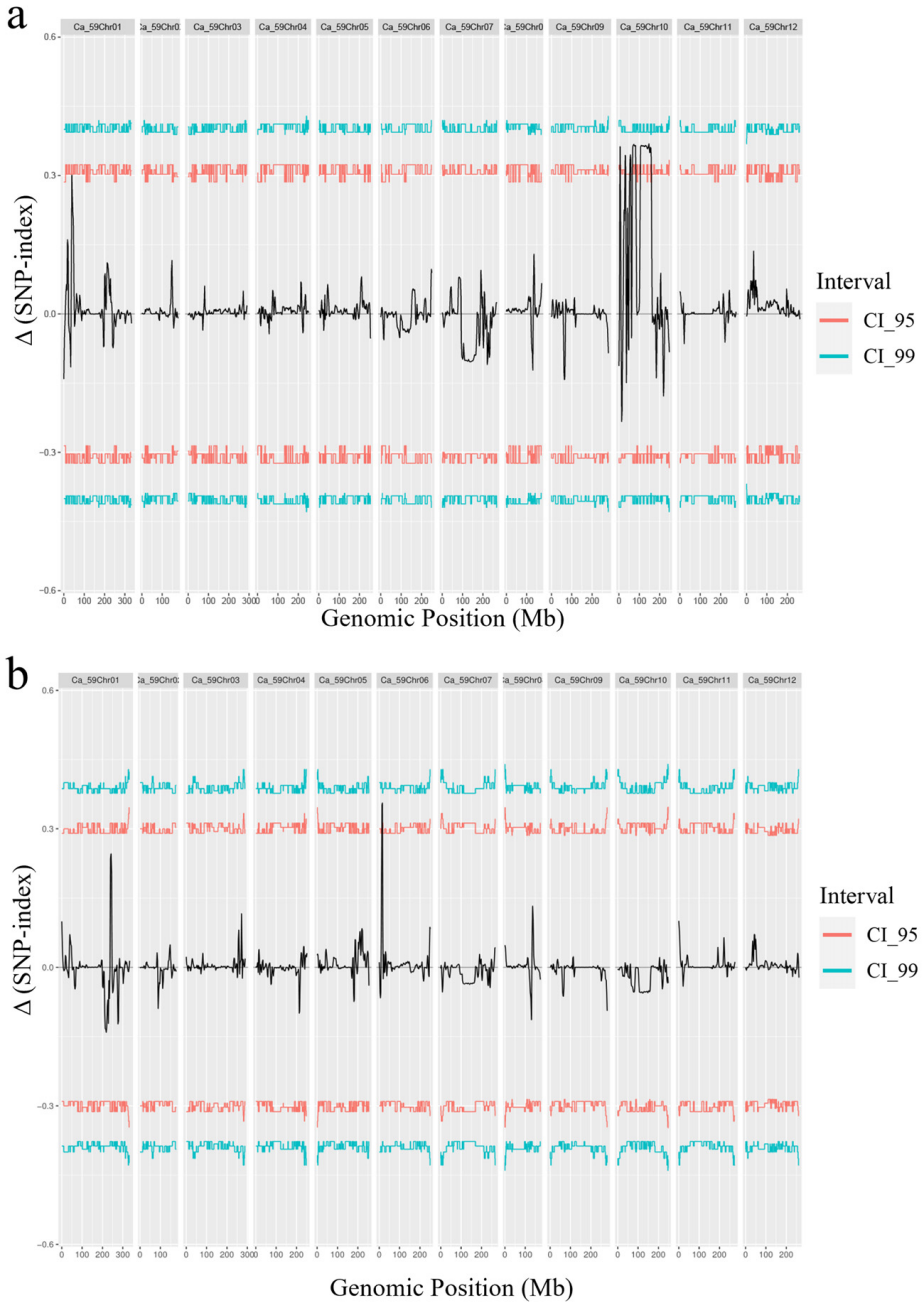
Distribution of Δ (SNP-index) on the pepper chromosome. **(A)** Distribution of Δ(SNP-index) on the pepper chromosome in the green fruit stage. **(B)** Distribution of Δ(SNP-index) on the pepper chromosome in the mature fruit stage. **(C)** Distribution of G’value on chromosomes in green fruit color stage. **(D)** Distribution of G’value on chromosomes in mature fruit color stage, the red line is the threshold line.

The parental lines and an F_2_ population of green and pale green fruits were subjected to whole-genome resequencing using the reference genome of the “CA59” pepper. A total of 540.18 GB of sequencing data was generated. A comprehensive set of 26,581,892 SNPs was identified in the green pepper population. After rigorous quality control, 14,230,899 high-quality SNPs were selected for subsequent quantitative trait loci (QTL) analysis. By calculating the SNP-index values and Gprime values using the high-quality SNPs derived from the green and pale green pools and subsequently calculating the difference to obtain Δ(SNP-index) values, two peaks were observed on Chr10 and Chr1 in the Δ(SNP-index) distribution plot. The peak of Chr10 spanned from 7,799 bp to 203,707,926 bp, covering a length of 203.7 Mb and encompassing 1,782 predicted genes. The highest Gprime value within this region was observed at 5,833,414 bp, reaching 38.37. Consequently, this QTL was designated as *gyqtl10.1*. The peak of Chr1 spanned from 59,369 bp to 57,830,451 bp, with a length of 57.77 Mb containing 1,757 predicted genes. The highest Gprime value within this region was located at 15,671,853 bp, reaching 44.35. Hence, this QTL was named *gyqtl1.1* (**Figures 3A, C**).

The parental lines and an F_2_ population of red and orange fruits were subjected to whole-genome resequencing using the reference genome of “CA59” pepper. A total of 529.08 Gb of sequencing data was generated, enabling the detection of single nucleotide polymorphisms (SNPs) and short insertion-deletion mutations (InDels) within the population. A comprehensive set of 26,325,289 SNPs was identified in the mature pepper population. After rigorous quality control, 17,365,545 high-quality SNPs were selected for subsequent quantitative trait loci (QTL) analysis. By calculating the SNP-index values and Gprime values using the high-quality SNPs derived from the red and orange pools and subsequently calculating the difference to obtain Δ(SNP-index) values, a distinctive peak was observed on Chr6 in the Δ(SNP-index) distribution plot. This peak spanned from 84 bp to 502,680,735 bp, covering a length of 50.27 Mb and encompassing 1,950 predicted genes. The highest Gprime value within this region was observed at 5,833,414 bp, reaching 30.77. Consequently, this QTL was designated as *roqtl6.1.* A potential minor-effect peak was identified on Chr1, spanning from 209,584,520 bp to 253,781,004 bp, with a length of 44.2 Mb containing 541 predicted genes. The highest Gprime value within this region was located at 242,131,481 bp, reaching 6.0. Hence, this QTL was named *roqtl1.1* (**Figures 3B, D**).

### 3.5 Construction of genetic linkage maps for pepper fruit color using InDel markers

**Figure 4:**
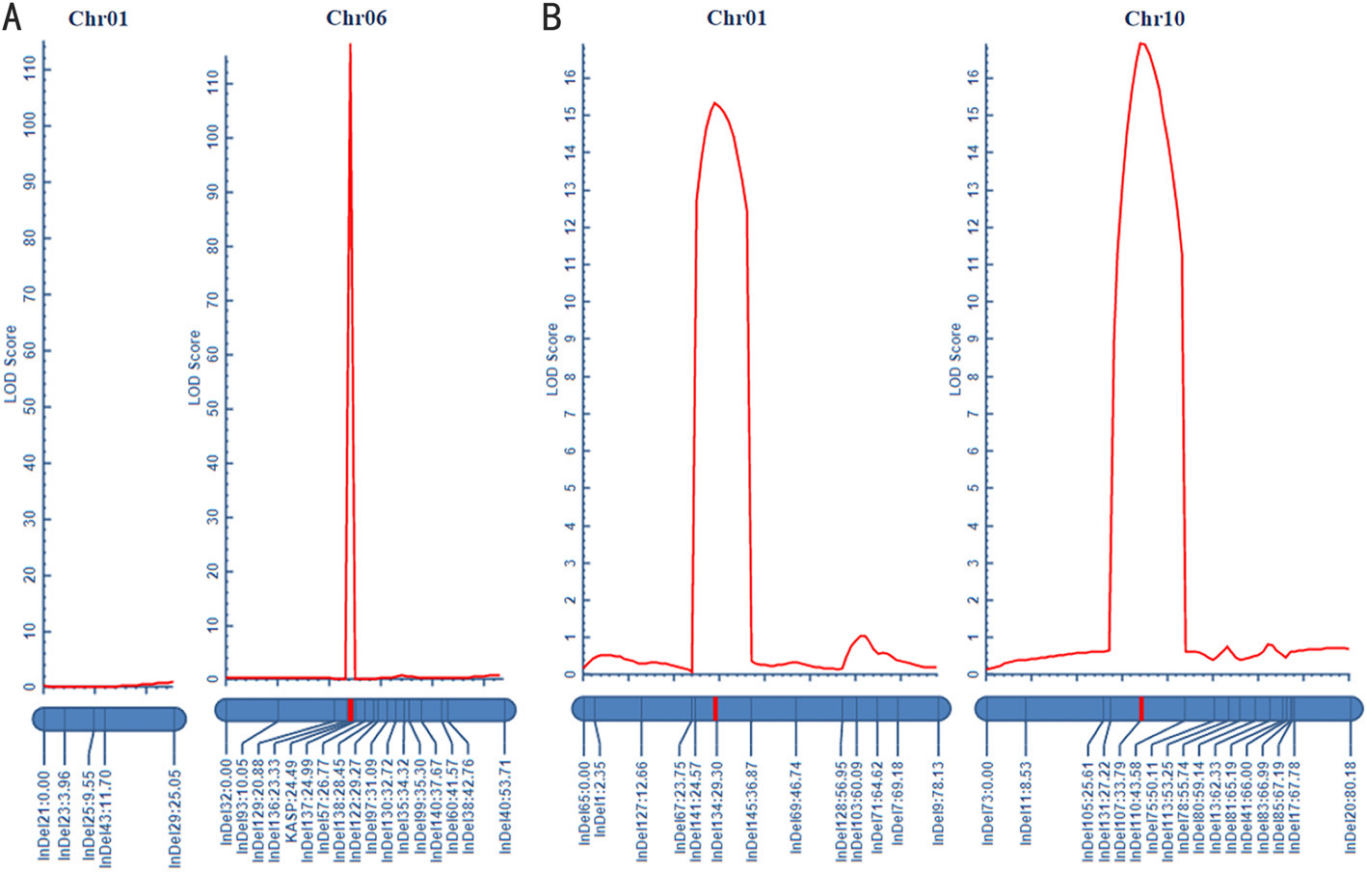
Genetic linkage mapping of two fruit colors in pepper. **(A)** Genetic linkage mapping of mature fruit color. **(B)** Genetic linkage mapping of green fruit color.

In this study, the candidate intervals for green fruit color were initially located on Chr10 and 1. To further narrow the mapping intervals, two parental lines (D24 and D47), F_1_ progeny, and 259 F_2_ individuals were used as templates for deep re-sequencing. By exploiting the polymorphism nature of InDel markers in the F_2_ population, a genetic linkage map was constructed using IciMapping software. Thirteen polymorphic InDel markers were selected to construct the genetic linkage map on Chr1 (**Supplementary Table 1**). The total genetic distance of the constructed map was 78.13 cm, with the linkage interval between InDel141 and InDel134 corresponding to a physical distance of 2.75 Mb (14,372,013 bp-17,126,499 bp). The maximum LOD value was 15.29, and the phenotypic variance explained (PVE) of the identified QTL was 19.62%. Similarly, seventeen polymorphic InDel markers were chosen to construct the genetic linkage map on Chr10 (**Supplementary Table 13**). The total genetic distance covered by this map was 80.18 cm, with the linkage interval located between InDel107 and InDel110, corresponding to a physical distance of 3.38 Mb (8,249,029 bp-11,626,729 bp). The maximum LOD value obtained was 16.9089, and the PVE of this QTL was 22.09% (**Supplementary Table 13**; **Figure 4A**).

The preliminary mapping intervals for mature fruit color in Capsicum were determined to be located on Chr6 and Chr1. For Chr1, five InDel markers were screened within the mapping interval, but no linkage was observed, indicating that Chr1 is not associated with mature fruit color in pepper. On the other hand, sixteen polymorphic InDel markers and one KASP marker were selected to construct the genetic linkage map on Chr6 (**Supplementary Table 4**). The total genetic distance covered by this map was 53.71 cm, with the linkage interval between InDel136 and KASP corresponding to a physical distance of 153.49 kb (9,563,662 bp-9,717,156 bp). The LOD value reached a maximum of 115.05, and the contribution rate of this QTL was 81.70% (**Supplementary Table 14**; **Figure 4B**).

### 3.6 Analysis of candidate genes for fruit color-related mapping intervals

Genetic linkage analysis was conducted to determine the intervals of genetic linkage associated with the characteristic. The gene function annotation results were produced by comparing the candidate genes within these intervals using Diamond and BLAST tools. The results showed that a total of 115 genes (116 transcripts) were found in the candidate region of chromosome *gyqtl1.1* linked to pepper of which the NT database registered 113 genes (114 transcripts) and NR (**Supplementary Table 15**). Additionally, within the putative region of Chr10 *gyqtl10.1* associated with green fruit; 87 genes were annotated as shown in **Supplementary Table 16**. It is worth noting that all 87 genes were successfully annotated in both the NR and NT databases. Based on gene function annotation and previously published related gene information, we have identified the *GLK2* (*Capann_59V1aChr10g003610*) gene as a potential candidate gene in the linkage interval of Chr10 (**Supplementary Table 17**). This gene may play a crucial role in controlling the green peel color.

The CDS sequences of 17 candidate genes in the candidate region were aligned to NT, NR, and Swisprot databases for functional annotation using Diamond and BLAST software. The findings indicated that all genes within the *roqtl6.1* candidate area of Chr6 in pepper are documented and included in **Supplementary Table 18**. A capsanthin/capsanthin synthetase gene, *CapCCS* (*Capann_59V1aChr06g006200*), was discovered by searching for known color-related pepper ripening genes. The difference in pepper ripening color features within this population may be primarily attributed to this gene (**Supplementary Table 19**). Previous research has indicated that three loci primarily regulate the variation in ripe fruit color in peppers. These loci are the *C1* locus, which is located on Chr1 and encodes the *PRR2* gene (Jeong et al., 2020); the *C2* locus, which is on Chr4 and encodes the *PSY1* gene (Huh et al., 2001); and the *Y* locus on chr6encoding the *CCS* gene (Lefebvre et al., 1998). Based on the findings, it was hypothesized that the *CapCCS* gene, present in the candidate interval on Chr6, could be the primary gene responsible for the variation in mature fruit color from red to orange or orange to red.

### 3.7 Sequence analysis of candidate gene *CapGLK2* for green fruit color

**Figure 5:**
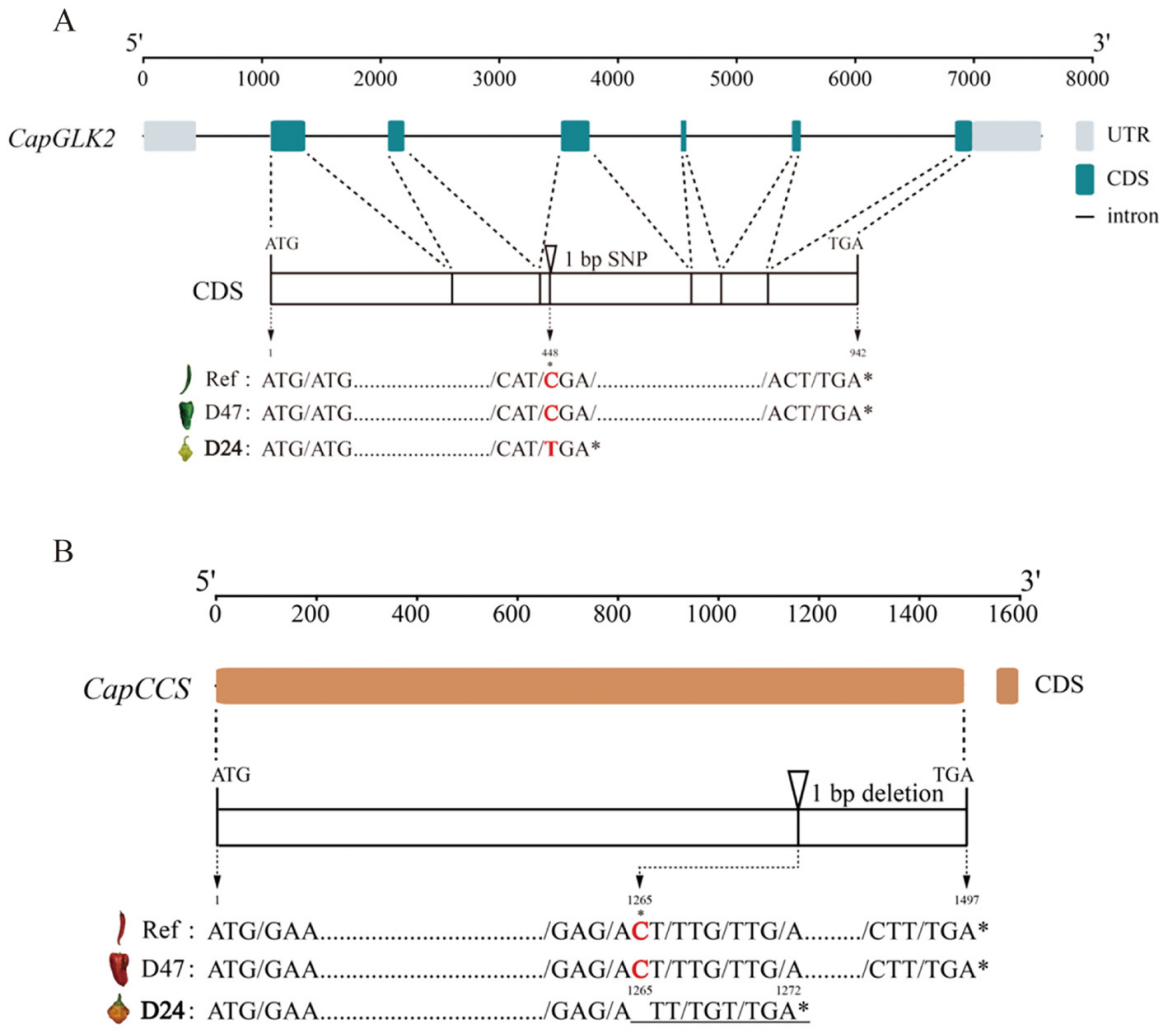
Sequence analysis map of key candidate genes. **(A)** The sequence analysis of the key candidate gene *CapGLK2* in *gyqtl10.1*. **(B)** The sequence analysis of the key candidate gene *CapCCS* in *roqtl6.1*. Ref indicates the reference genome of “CA59” chili peppers, and ref, D47, and D24 are the sequence variation diagrams of the candidate genes in the three materials, respectively. Ref indicates the reference genome of “CA59” chili peppers, and Ref, D47, and D24 are the nucleic acid variants of the candidate genes in these three materials. * denotes significance difference.

The *CapGLK2* gene is positioned on Chr10 between positions 9,177,688 bp and 9,185,257 bp. This gene consists of six exons with a coding length of 942 bp. Using genomic resequencing, we detected an SNP at position 448 bp within the coding region of *CapGLK2* in the D24 parental line. The SNP resulted in a C to T base substitution. Our analysis using open reading frame (ORF) prediction on the altered coding sequence in the D24 parent revealed that this SNP causes *CapGLK2* to produce a stop codon, which in turn causes the coding sequence to terminate prematurely. Notably, the premature transcriptional termination caused by the SNP could contribute to the pale green color phenotype seen in young pepper fruits (**Figure 5A**).

**Figure 6:**
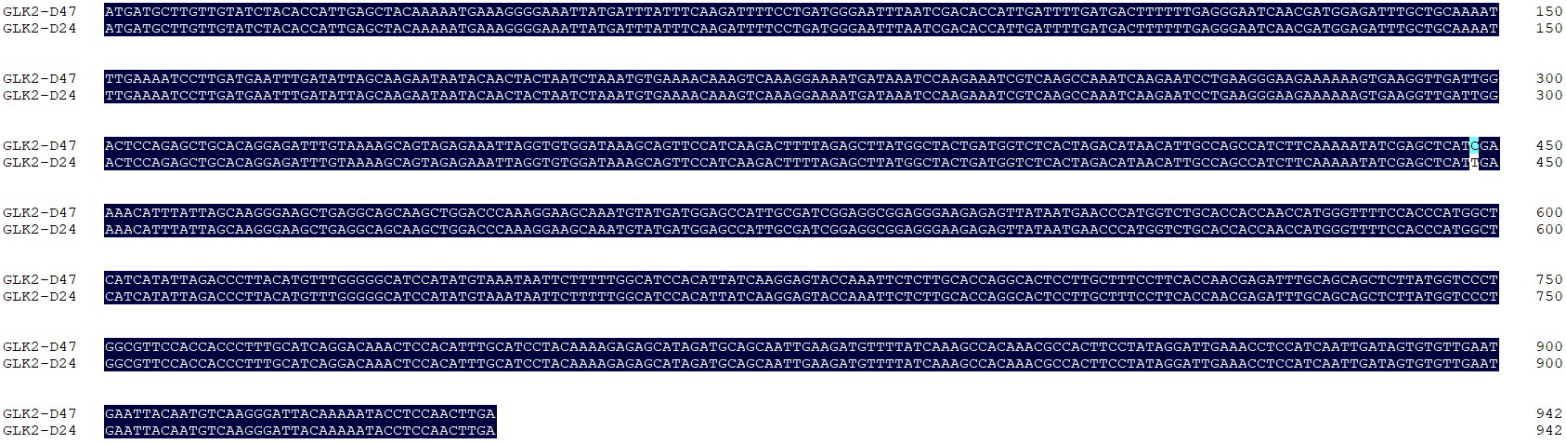
Comparison of CDS sequences of *CapGLK2* gene in D24 and D47. *GLK2*-D47 and *GLK2*-D24 are the CDS sequences of the *CapGLK2* gene in the D47 and D24 materials, respectively.

To further confirm the presence of the SNP mutation in the *CapGLK2* gene sequence, we extracted genomic RNA from the green fruit of pepper lines D24 and D47 and used its cDNA as templates for PCR amplification. The PCR products were purified, sequenced, and aligned against the reference genome. We found that the *CapGLK2* gene showed the same mutation as the above sequence analysis at 488bp between the CDS sequences of D47 and D24 (**Figure 6**). These findings provide further evidence supporting the role of the *CapGLK2* gene as a key regulator of fruit color in pepper.

**Figure 7:**
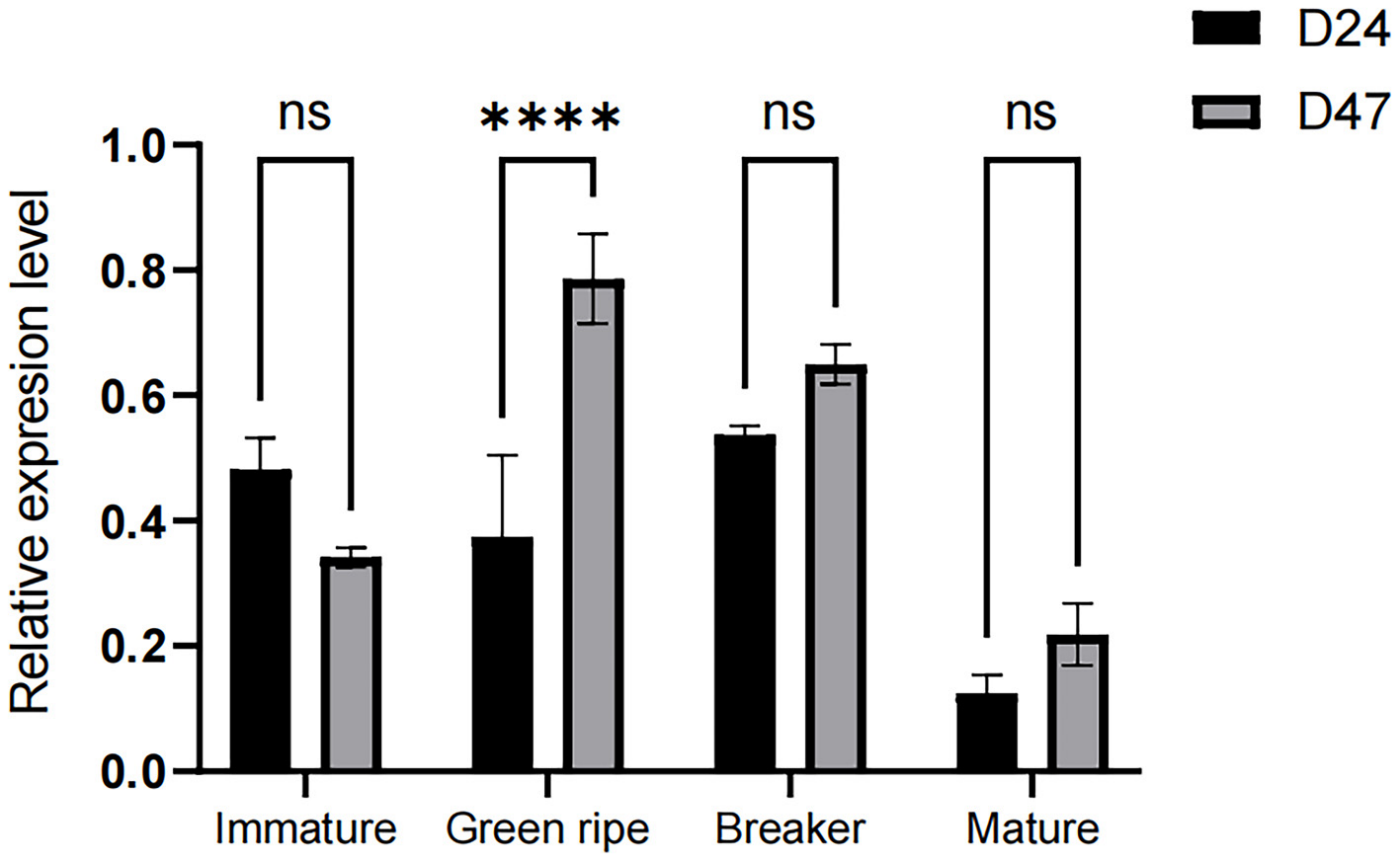
Expression of *CapGLK2* gene in different growth periods of pepper fruits The **** in the figure indicates a significant difference at the 0.01 significance level, i.e., a highly significant difference.

Expression analysis showed that the *CapGLK2* gene was not expressed during immature, breaker, and mature but displayed dominant expression at the green ripe stage. (**Figure 7**)

### 3.8 Cloning and expression analysis of CapCCS associated with mature fruit color

The *CapCCS* gene is located on Chr6 of the “CA59” pepper genome between positions 9,715,892 bp and 9,717,388 bp. Genomic resequencing revealed an InDel in the coding region of *CapCCS* in the D24 parent line at position 1,265 bp, deleting a single nucleotide C. In the D24, ORF prediction identified a frameshift mutation in the *CapCCS* gene, causing premature termination at 1,272 bp. Phenotypic analysis showed that the D24 parent line, with the InDel mutation, exhibited orange mature fruit color, while the D47 parent line and the reference genome CA59, lacking the InDel mutation, displayed red mature fruit color (**Figure 5B**). These findings suggest that the identified InDel may be responsible for the orange mature fruit color in D24.

**Figure 8:**
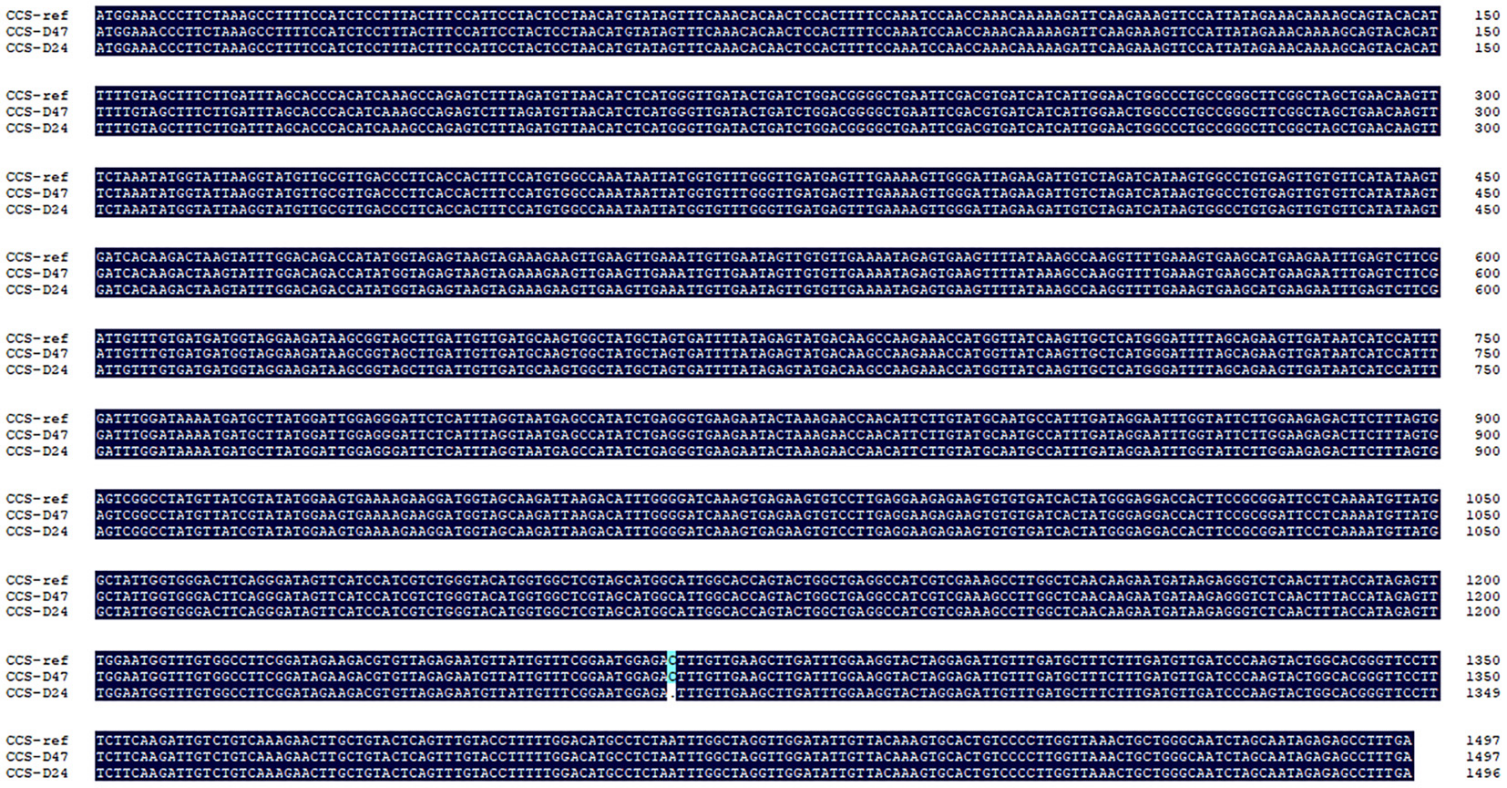
Nucleic acid sequence comparison of *CapCCS* gene in D24 and D47, and the reference genome *CapCCS*. CCS-ref is the nucleic acid sequence of the *CapCCS* gene in the reference genome “CA59” chili peppers, and *CCS*-D47 and *CCS*-D24 are the nucleic acid sequences of the *CapCCS* gene in the D47 and D24 materials, respectively.

To further confirm the presence of the InDel mutation in the *CapCCS* gene sequence, we extracted genomic DNA from the young leaves of pepper lines D24 and D47 and used them as templates for PCR amplification (**Supplementary Figure 2**). The PCR products were purified, sequenced, and aligned against the reference genome. We found that the DNA sequence of *CapCCS* of D47 was identical to that of *CapCCS*, the reference genome of “CA59” pepper with red color of mature fruit. Contrarily, the reference genome “CA59” and D47 of the pepper were compared to the DNA sequence of *CapCCS* of the orange-colored D24 pepper, showing unidentical result (**Figure 8**). These findings provide further evidence supporting the role of the *CapCCS* gene as a key regulator of fruit color in pepper.

**Figure 9:**
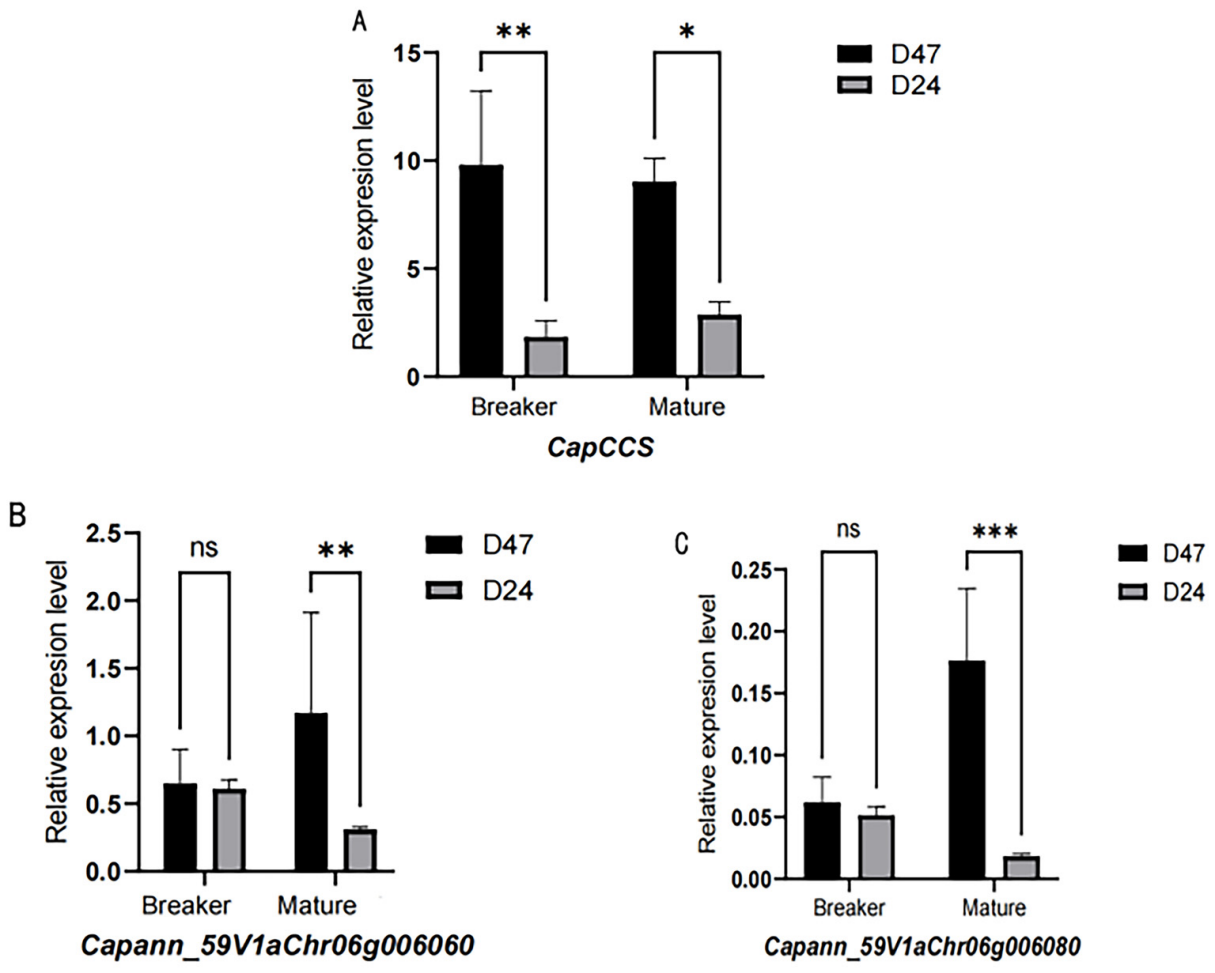
Expression of candidate genes during the breaker and mature stages of pepper fruits. **(A)** Expression of *CapCCS* gene during the breaker and mature stages of pepper fruits. **(B)** Expression of *Capann_59V1aChr06g006060* gene during the breaker and mature stages of pepper fruits. **(C)** Expression of *Capann_59V1aChr06g006080* gene during the breaker and mature stages of pepper fruits. The ** in the figure indicates a significant difference at the 0.01 significance level, i.e., a highly significant difference. * denotes significance difference.

Expression analysis showed that the *CapCCS* gene was not expressed during immature and commercial ripe stages but displayed dominant expression at the breaker stage. Expression analysis showed that *Capann_59V1aChr06g006060* and *Capann_59V1aChr06g006080* were not expressed during immature, green ripe, and breaker but displayed dominant expression at the mature stage (**Figure 9**).

## 4 Discussion

Chlorophyll is the main pigment responsible for peppers’ green fruit color. Still, other pigments, such as carotenoids and anthocyanins, can cause peppers’ fruits to turn purple (Liu et al., 2020) and black (Lightbourn et al., 2008), respectively. Segregation analysis conducted by using light yellow and green pepper materials suggested that the light-yellow color is controlled by a single recessive gene (Song et al., 2022). On the other hand, genetic analysis showed that a single locus dominant hereditary feature controls the light green color of green fruits (Wu et al., 2022). In this study, we employed a visual grading method to analyze fruit color as a qualitative trait, and our segregation analysis in the F_2_ population revealed a segregation ratio of green: yellowish green: pale green = 12:3:1, indicating that two pairs of alleles control fruit color in green fruit (**Figures 1A, C**). The green allele dominates epistatic effects over the yellowish-green allele. In contrast, yellowish green dominates the recessive pale green allele, suggesting two pairs of recessive homozygous genes regulate pale green peel characteristics.

The green fruit color in pepper is primarily regulated by the *pc1* and *pc10.1* loci, as revealed by genetic mapping studies (Brand et al., 2012; Borovsky et al., 2019). Previous studies identified *CaGLK2* as a candidate gene at the *pc10.1* locus, which controls natural variation in chlorophyll content and green fruit color by regulating chloroplast size (Brand et al., 2014). Genetic mapping using light green and green fruits identified *CaPP2C35* (*Capana10g001710*) as a candidate gene involved in regulating the accumulation of chlorophyll content in the peel (Wu et al., 2022), thereby influencing the formation of light green fruits. In this study, we performed genetic mapping using pale green and green fruits and identified two candidate intervals, *gyqtl1.1* and gyqtl10.1 (**Figure 3**). The *gyqtl10.1* interval was located Chr10 between 8.25 Mb and 11.63 Mb, containing 87 genes. *CapGLK2*, a candidate gene for fruit color regulation, was found within this interval. Green fruit peel from D24 and D47 parents showed differential expression of the *CapGLK2* gene. A pale green fruit peel from the D24 parent had an SNP that caused transcription to terminate prematurely due to the creation of a stop codon (**Figure 5A**). This data supports prior research suggesting *CapGLK2* as a critical candidate gene in the *gyqtl10.1* range (Brand et al., 2014). The *gyqtl1.1* interval was located on Chr1 between 14.37 Mb and 17.13 Mb, containing 115 genes (**Figure 4A**, **Supplementary Table 15**). Given its proximity to previous studies (Borovsky et al., 2019; Wu et al., 2022), it is speculated that other genes influencing green fruit color in pepper may be present within the *gyqtl1.1* interval, although further validation is required.

The mature fruit color includes red, orange, and yellow colors. It is widely believed that the genetic control of red and orange mature fruit color in peppers follows a simple Mendelian inheritance pattern (Huh et al., 2001; Kim et al., 2010). In this study, the F_2_segregating population of red-fruited (D47) and orange-fruited (D24) peppers was visually observed, and the ratio of red to orange fruits approximately followed a 3:1 segregation ratio, confirming the control of ripe fruit color in peppers by a single gene pair (**Figures 1B, C**). Hurtado-Hernandez and Smith proposed that mature fruit color in peppers is mainly controlled by three loci: *C1*, *C2*, and *Y* (Hurtado-Hernandez and Smith, 1985). Subsequent studies identified the *PRR2* gene on Chr1 (Jeong et al., 2020; Lee et al., 2020) the *PSY1* gene on Chr4 (Huh et al., 2001), and the *CCS* gene on Chr6 (Popovsky and Paran, 2000; Lefebvre et al., 1998) as the candidate genes for these three loci, respectively. *PRR2* gene silencing produces white-colored ripe fruits (Jeong et al., 2020). In addition, the *PSY1* gene encodes lycopene synthase involved in carotenoid synthesis, and the *CCS* gene is the pepper capsanthin-capsorubin synthase gene. In our study, through BSA-seq and constructing a genetic linkage map, we identified a region, *roqtl6.1*, associated with red and orange fruit colors (**Figure 3**). The physical position of this linked region is located on Chr6, spanning from 9,563,662 bp to 9,717,156 bp, with a size of 153.49 kb and containing 17 candidate genes that align with the *Y* locus. Functional annotation of these candidate genes revealed that the *CCS* gene, a candidate gene for the *Y* locus, is located within the *roqtl6.1* linked region. Hence, we hypothesize that the *CapCCS* gene is the candidate gene responsible for determining mature fruit color.

The *CCS* gene is one of the key genes that regulate the color of mature peppers. Variations and deletions in the *CCS* gene can result in orange or yellow mature fruit color (Popovsky and Paran, 2000;Lefebvre et al., 1998). However, the loss of the *CCS* gene is not the sole cause of yellow fruit color. Having detected the presence of the *CCS* gene in yellow peppers, an 8 bp insertion at position 1,431 bp and a single base mutation at position 599 bp in the coding region of *CCS* resulted in premature termination of its transcription (Ha et al., 2007), leading to yellow mature fruit color. In our experiment, we found a deletion of a C base at position 1,265 bp in the coding region of the *CapCCS* gene in the yellow mature fruit of D24, resulting in premature termination of the *CapCCS* gene translation, (**Figure 8**). This deletion of the C base co-segregated with the yellow fruit phenotype. Furthermore, the promoter region of *CapCCS* was identical in the red mature fruit parent D47 and the yellow mature fruit parent D24. These findings suggest that the disparities in the red and orange mature fruit color within our experimental population are attributed to polymorphisms in the coding sequence of the *CapCCS* gene, resulting in premature termination of translation.

## 5 Conclusions

Our study determines the inheritance of green and mature pepper fruit colors independently. The green and pale green colors of premature pepper fruits are influenced by two quantitative trait loci (QTLs) located on Chr1 (*gyqtl1.1*) and Chr10 (*gyqtl10.1*), respectively. We have identified the *CapGLK2* gene within the *gyqtl10.1* region and suspect that an SNP mutation may cause the green to pale green fruits. Furthermore, in our experimental population, the color of mature pepper fruits is controlled by one pair of recessive alleles. The gene *CapCCS* involved is found within the potential region *roqtl6.1* on Chr6. A single base deletion of a C at position 1,265 bp within the coding region of the *CapCCS* gene causes premature termination of translation, giving the mature fruits an orange color.

## Data Availability

The original contributions presented in the study are publicly available. This data can be found here: NCBI, BankIt2876008 PQ422120, BankIt2876008 PQ422121, BankIt2876008 PQ422122, BankIt2876008 PQ422123, BankIt2876008 PQ422124, BankIt2876008 PQ422125, BankIt2876008 PQ422126, BankIt2876008 PQ422127, BankIt2876008 PQ422128, BankIt2876008 PQ422129, BankIt2876008 PQ422130, BankIt2876008 PQ422131, BankIt2876008 PQ422132, BankIt2876008 PQ422133, BankIt2876008 PQ422134, BankIt2876008 PQ422135, BankIt2876008 PQ422136, BankIt2876008 PQ422137, BankIt2876008 PQ422138, BankIt2876008 PQ422139, BankIt2876008 PQ422140, BankIt2876008 PQ422141, BankIt2876008 PQ422142.

## References

[B1] Allen G. C. Flores-Vergara M. A. Krasynanski S. Kumar S. Thompson W. F. (2006). A modified protocol for rapid DNA isolation from plant tissues using cetyltrimethylammonium bromide. Nat. Protoc. 1, 2320–2325. doi: 10.1038/nprot.2006.384 17406474

[B2] Altschul S. F. Gish W. Miller W. Myers E. W. Lipman D. J. (1990). Basic local alignment search tool. J. Mol. Biol. 215, 403–410. doi: 10.1006/jmbi.1990.9999 2231712

[B3] Alvarez-Parrilla E. de la Rosa L. A. Amarowicz R. Shahidi F. (2011). Antioxidant activity of fresh and processed Jalapeno and Serrano peppers. J. Agric. Food Chem. 59, 163–173. doi: 10.1021/jf103434u 21126003

[B4] Borovsky Y. Monsonego N. Mohan V. Shabtai S. Kamara I. Faigenboim A.. (2019). The zinc-finger transcription factor CcLOL1 controls chloroplast development and immature pepper fruit color in Capsicum chinense and its function is conserved in tomato. Plant J. 99, 41–55. doi: 10.1111/tpj.14305 30828904

[B5] Brand A. Borovsky Y. Hill T. Rahman K. A. A. Bellalou A. Van Deynze A.. (2014). *CaGLK2* regulates natural variation of chlorophyll content and fruit color in pepper fruit. Theor. Appl. Genet. 127, 2139–2148. doi: 10.1007/s00122-014-2367-y 25096887

[B6] Brand A. Borovsky Y. Meir S. Rogachev I. Aharoni A. Paran I. (2012). *pc8. 1*, a major QTL for pigment content in pepper fruit, is associated with variation in plastid compartment size. Planta 235, 579–588. doi: 10.1007/s00425-011-1530-9 21987007

[B7] Buchfink B. Reuter K. Drost H. G. (2021). Sensitive protein alignments at tree-of-life scale using DIAMOND. Nat. Methods 18, 366–368. doi: 10.1038/s41592-021-01101-x 33828273 PMC8026399

[B8] Chen Y. Chen Y. Shi C. Huang Z. Zhang Y. Li S.. (2018). SOAPnuke: a MapReduce acceleration-supported software for integrated quality control and preprocessing of high-throughput sequencing data. Gigascience 7, gix120. doi: 10.1093/gigascience/gix120 29220494 PMC5788068

[B9] Chen C. Chen H. Zhang Y. Thomas H. R. Frank M. H. He Y.. (2020). TBtools: an integrative toolkit developed for interactive analyses of big biological data. Mol. Plant 13, 1194–1202. doi: 10.1016/j.molp.2020.06.009 32585190

[B10] Cingolani P. Platts A. Wang L. L. Coon M. Nguyen T. Wang L.. (2012). A program for annotating and predicting the effects of single nucleotide polymorphisms, SnpEff: SNPs in the genome of Drosophila melanogaster strain w1118; iso-2; iso-3. fly 6, 80–92. doi: 10.4161/fly.19695 22728672 PMC3679285

[B11] Ha S. H. Kim J. B. Park J. S. Lee S. W. Cho K. J. (2007). A comparison of the carotenoid accumulation in Capsicum varieties that show different ripening colours: deletion of the capsanthin-capsorubin synthase gene is not a prerequisite for the formation of a yellow pepper. J. Exp. Bot. 58, 3135–3144. doi: 10.1093/jxb/erm132 17728301

[B12] Huh J. H. Kang B. C. Nahm S. H. Kim S. Ha K. S. Lee M. H.. (2001). A candidate gene approach identified phytoene synthase as the locus for mature fruit color in red pepper (*Capsicum* spp.). Theor. Appl. Genet. 102, 524–530. doi: 10.1007/s001220051677

[B13] Hurtado-Hernandez H. Smith P. G. (1985). Inheritance of mature fruit color in *Capsicum annuum* L. J. Heredity 76, 211–213. doi: 10.1093/oxfordjournals.jhered.a110070

[B14] Jang S. J. Jeong H. B. Jung A. Kang M. Y. Kim S. Ha S. H.. (2020). Phytoene synthase 2 can compensate for the absence of *PSY1* in the control of color in Capsicum fruit. J. Exp. Bot. 71, 3417–3427. doi: 10.1093/jxb/eraa155 32219321 PMC7475241

[B15] Jeong H. B. Jang S. J. Kang M. Y. Kim S. Kwon J. K. Kang B. C. (2020). Candidate gene analysis reveals that the fruit color locus *C1* corresponds to *PRR2* in pepper (*Capsicum frutescens*). Front. Plant Sci. 11, 399. doi: 10.3389/fpls.2020.00399 32328078 PMC7161348

[B16] Kim O. R. Cho M. C. Kim B. D. Huh J. H. (2010). A splicing mutation in the gene encoding phytoene synthase causes orange coloration in Habanero pepper fruits. Molecules Cells 30, 569–574. doi: 10.1007/s10059-010-0154-4 21120629

[B17] Kormos J. (1960). Die Genetischen Typen der Carotinoid-Systeme der Parpikafrucht. Acta Bot.(Acad. Sci. Hung.) 6, 305–319. doi: 10.1556/verb.6.2004.2.9

[B18] Lee S. B. Kim J. E. Kim H. T. Lee G. M. Kim B. S. Lee J. M. (2020). Genetic mapping of the *c1* locus by GBS-based BSA-seq revealed *Pseudo-Response Regulator 2* as a candidate gene controlling pepper fruit color. Theor. Appl. Genet. 133, 1897–1910. doi: 10.1007/s00122-020-03565-5 32088729

[B19] Lefebvre V. Kuntz M. Camara B. Palloix A. (1998). The capsanthin-capsorubin synthase gene: a candidate gene for the *y* locus controlling the red fruit colour in pepper. Plant Mol. Biol. 36, 785–789. doi: 10.1023/a:1005966313415 9526511

[B20] Li H. Durbin R. (2009). Fast and accurate short read alignment with Burrows-Wheeler transform. bioinformatics 25, 1754–1760. doi: 10.1093/bioinformatics/btp324 19451168 PMC2705234

[B21] Li H. Handsaker B. Wysoker A. Fennell T. Ruan J. Homer N.. (2009). The sequence alignment/map format and SAMtools. Bioinformatics 25, 2078–2079. 19505943 10.1093/bioinformatics/btp352 PMC2723002

[B22] Liao Y. Wang J. Zhu Z. Liu Y. Chen J. Zhou Y.. (2022). The 3D architecture of the pepper genome and its relationship to function and evolution. Nat. Commun. 13, 3479. doi: 10.1038/s41467-022-31112-x 35710823 PMC9203530

[B23] Lightbourn G. J. Griesbach R. J. Novotny J. A. Clevidence B. A. Rao D. D. Stommel J. R. (2008). Effects of anthocyanin and carotenoid combinations on foliage and immature fruit color of *Capsicum annuum* L. J. heredity 99, 105–111. doi: 10.1093/jhered/esm108 18222931

[B24] Liu Y. Lv J. Liu Z. Wang J. Yang B. Chen W.. (2020). Integrative analysis of metabolome and transcriptome reveals the mechanism of color formation in pepper fruit (*Capsicum annuum* L.). Food Chem. 306, 125629. doi: 10.1016/j.foodchem.2019.125629 31629298

[B25] Liu Z. Yang B. Huang R. Suo H. Zhang Z. Chen W.. (2022). Transcriptome-and proteome-wide association of a recombinant inbred line population revealed twelve core QTLs for four fruit traits in pepper (*Capsicum annuum* L.). Horticulture Res. 9, uhac015. doi: 10.1093/hr/uhac015 PMC9016867 35147182

[B26] Mansfeld B. N. Grumet R. (2018). QTLseqr: An R package for bulk segregant analysis with next-generation sequencing. Plant Genome 11, 180006. 10.3835/plantgenome2018.01.0006 PMC12810111 30025013

[B27] Martí M. C. Camejo D. Olmos E. Sandalio L. M. Fernández-García N. Jiménez A.. (2009). Characterisation and changes in the antioxidant system of chloroplasts and chromoplasts isolated from green and mature pepper fruits. Plant Biol. 11, 613–624. doi: 10.1111/j.1438-8677.2008.00149.x 19538399

[B28] Pan Y. Bradley G. Pyke K. Ball G. Lu C. Fray R.. (2013). Network inference analysis identifies an *APRR2-like* gene linked to pigment accumulation in tomato and pepper fruits. Plant Physiol. 161, 1476–1485. doi: 10.1104/pp.112.212654 23292788 PMC3585610

[B29] Popovsky S. Paran I. (2000). Molecular genetics of the *y* locus in pepper: its relation to capsanthin-capsorubin synthase and to fruit color. Theor. Appl. Genet. 101, 86–89. doi: 10.1007/s001220051453

[B30] Qin C. Yu C. Shen Y. Fang X. Chen L. Min J.. (2014). Whole-genome sequencing of cultivated and wild peppers provides insights into Capsicum domestication and specialization. Proc. Natl. Acad. Sci. 111, 5135–5140. doi: 10.1073/pnas.1400975111 24591624 PMC3986200

[B31] Song Z. Zhong J. Dong J. Hu F. Zhang B. Cheng J.. (2022). Mapping immature fruit colour-related genes via bulked segregant analysis combined with whole-genome re-sequencing in pepper (*Capsicum annuum*). Plant Breed. 141, 277–285. doi: 10.1111/pbr.12997

[B32] Takagi H. Abe A. Yoshida K. Kosugi S. Natsume S. Mitsuoka C.. (2013). QTL-seq: rapid mapping of quantitative trait loci in rice by whole genome resequencing of DNA from two bulked populations. Plant J. 74, 174–183. doi: 10.1111/tpj.12105 23289725

[B33] Wahyuni Y. Ballester A. R. Sudarmonowati E. Bino R. J. Bovy A. G. (2011). Metabolite biodiversity in pepper (*Capsicum*) fruits of thirty-two diverse accessions: Variation in health-related compounds and implications for breeding. Phytochemistry 72, 1358–1370. doi: 10.1016/j.phytochem.2011.03.016 21514607

[B34] Wahyuni Y. Ballester A. R. Sudarmonowati E. Bino R. J. Bovy A. G. (2013). Secondary metabolites of *Capsicum* species and their importance in the human diet. J. Natural products 76, 783–793. doi: 10.1021/np300898z 23477482

[B35] Wellburn A. R. Lichtenthaler H. (1984). “Formulae and program to determine total carotenoids and chlorophylls a and b of leaf extracts in different solvents,” in Advances in Photosynthesis Research: Proceedings of the VIth International Congress on Photosynthesis, Brussels, Belgium, August 1–6, 1983 Volume 2. 9–12 (Brussels, Belgium: Springer Netherlands).

[B36] Wu L. Wang H. Liu S. Liu M. Liu J. Wang Y.. (2022). Mapping of *CaPP2C35* involved in the formation of light-green immature pepper (*Capsicum annuum* L.) fruits via GWAS and BSA. Theor. Appl. Genet. 135, 591–604. doi: 10.1007/s00122-021-03987-9 34762177

