# Supplementary material for: Mapping and cloning of pepper fruit color-related genes based on BSA-seq technology
Source: Front Plant Sci. 2024 Oct 25;15:1447805. doi: 10.3389/fpls.2024.1447805 (PMC11543483; doi:10.3389/fpls.2024.1447805)
Supplement: Supplementary file 1 [file DataSheet1.docx]

# Supplementary Figures


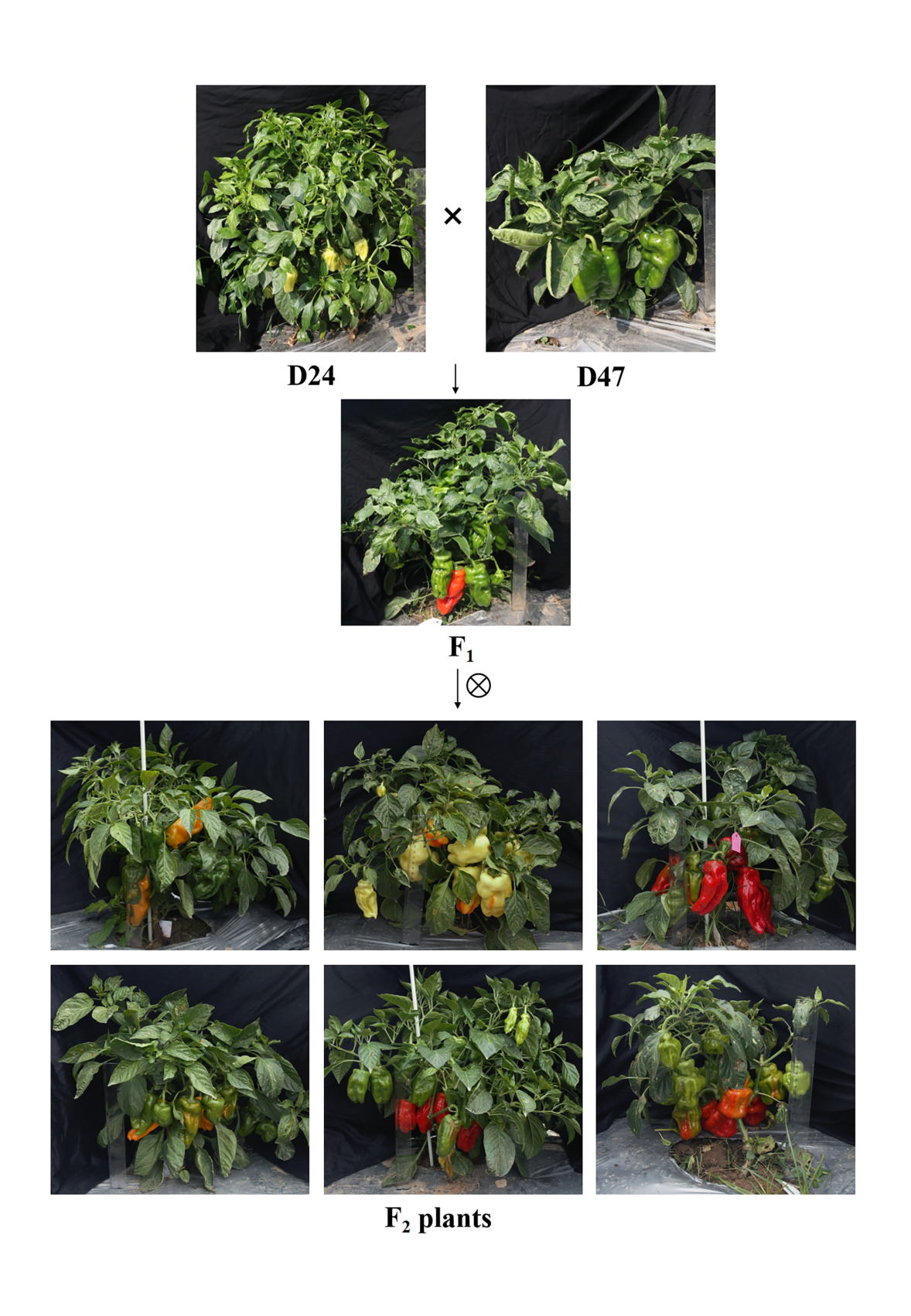


**Fig. S1** Phenotypic map of plants of the combination D24 × D47


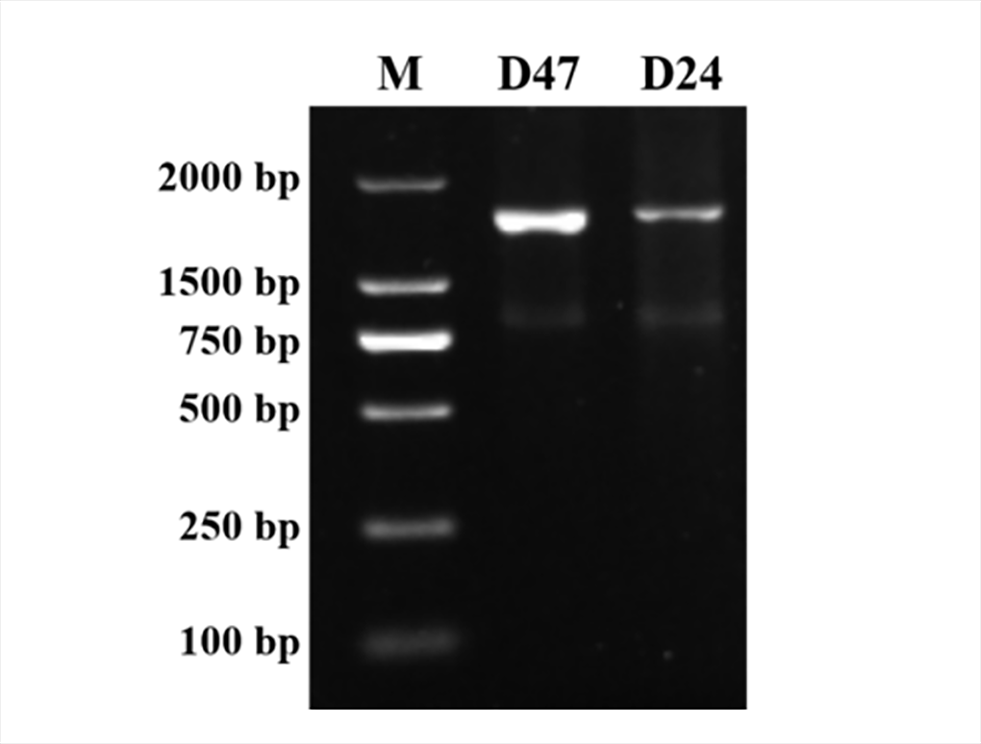


**Fig. S2** Amplified gel map of DNA sequences from *CapCCS.*M indicates DL2000 DNA Marker, D47 and D24 are DNA amplification gel plots of *CapCCS*, respectively.

# Supplementary Tables

Table S1. Primer information of the green fruit InDel marker Chr01positioning interval

| Primer name | Positioning interval | Physical location | sequence（5'→3'） |
| --- | --- | --- | --- |
| InDel65 | *gyqtl1.1* | 1,899,365 | F:GTTCCCTTTTTACTTGCC  R:TGGTTCCACCACTGACTG |
| InDel1 | *gyqtl1.1* | 2,707,892 | F:GATTTAAGATCAAGGGACGACC  R:AAACGACTCACTACAAAGGCTG |
| InDel127 | *gyqtl1.1* | 7,476,742 | F:ACCTACCAACCCCTTTCC  R:AAAAGAAGCACTAAATAATAAACT |
| InDel67 | *gyqtl1.1* | 13,277,559 | F:AGTTGGTCTCATATAAATCCCA  R:TTACTGTTTCTTGTCAGCGTTG |
| InDel141 | *gyqtl1.1* | 14,372,013 | F:TCCTTTTTGCAACAATATGACA  R:CCTTTTAGCTGGGTCCAATT |
| InDel134 | *gyqtl1.1* | 17,126,499 | F:ACCGCCACAACATCAAACA  R:CGAGGAGGAAGAGAAGACAAG |
| InDel145 | *gyqtl1.1* | 19,150,732 | F:TGAAAAATGGACTGGTTGGA  R:GCCTTCTAAAAGAGGTGTGGT |
| InDel69 | *gyqtl1.1* | 21,635,087 | F:TTGGTTGTTGTGTAGGTTATT  R:TGAGAGTCTATTTGTGTATTA |
| InDel128 | *gyqtl1.1* | 26,956,842 | F:TATTTTGTTGGATAACCGTGG  R:AGCTTCATACTGTGATCTGGA |
| InDel103 | *gyqtl1.1* | 29,447,239 | F:AAAGAACCCCTCTACGAAT  R:CCAACCCAGTGGACAAT |
| InDel71 | *gyqtl1.1* | 34,054,422 | F:CTTCAGTGTCCTTGACTACCA  R:AAAAATAGCATCCATTGACCT |
| InDel7 | *gyqtl1.1* | 44,797,693 | F:TAGTAGCGATGGATGCC  R:AAAGCCACAAAAAATAAATGAT |
| InDel9 | *gyqtl1.1* | 55,802,762 | F:GTGTTTTGTTCCTCTTTCG  R:GCTCCTCTTGATTCCATTT |

Table S2. Primer information of green fruit InDel marker Chr10 positioning interval

| Primer name | Positioning interval | Physical location | sequence（5'→3'） |
| --- | --- | --- | --- |
| InDel73 | *gyqtl10.1* | 748,012 | F:TGTCAAGTTTGGGGTCA  R:AGCACATGCAAGTCGGA |
| InDel11 | *gyqtl10.1* | 2,409,266 | F:CGATACAGGGTGGGTGATG  R:CTGAAGAAGGAACTGGGGAA |
| InDel105 | *gyqtl10.1* | 5,897,434 | F:GAGGTCACGAGTTCAAGCG  R:ATGTAGATAAAAGGTCATCCCAG |
| InDel131 | *gyqtl10.1* | 6,915,058 | F:GCTTTAGGGTTCGCTCTTG  R:CCTTTGGTTGCTTTGGCT |
| InDel107 | *gyqtl10.1* | 8,249,029 | F:GAGTTAGGCTCGTGTGTCA  R:AGGAAAATCCCTTTGTTTATA |
| InDel110 | *gyqtl10.1* | 11,626,729 | F:AATCTGACGTGCATTGTAGATC  R:TAAACCTTCCAAACTTGTGTCT |
| InDel75 | *gyqtl10.1* | 14,140,269 | F:TATACATGCCAATGGTAACCTT  R:CATCTTTCCTTTCTAACTCCTC |
| InDel113 | *gyqtl10.1* | 20,734,425 | F:CATTTCTGAGTTCAATCCAA  R:AATATCTTCGGCTTTAAGGT |
| InDel78 | *gyqtl10.1* | 26,473,015 | F:AGAGTCCTTTTGCCTTGTG  R:AACTTTATTCCATTTGCTACCT |
| InDel80 | *gyqtl10.1* | 38,784,615 | F:CATCCCTAACTTCTTATTCCG  R:TTTTGAGGCTATAGAGCATTC |
| InDel13 | *gyqtl10.1* | 50,849,619 | F:AACCCAACCACCATCAACTA  R:ATCTACCAAACCCCAACAAG |
| InDel81 | *gyqtl10.1* | 76,108,973 | F:GTTCAGGGGAATGGACAA  R:AATACCCACCAAATGATAGA |
| InDel41 | *gyqtl10.1* | 102,172,887 | F:ATCCGAAGATGGTTAGTAGT  R:TCCAATGAAATAGAATGAGA |

Continued Table S2. Primer information of the green fruit InDel marker Chr10 positioning interval

| Primer name | Positioning interval | Physical location | sequence（5'→3'） |
| --- | --- | --- | --- |
| InDel83 | *gyqtl10.1* | 102,403,877 | F:CTCTATGCCCTTCCATCAAC  R:GTCAATAAGACCCTAAAAAACT |
| InDel85 | *gyqtl10.1* | 127,001,857 | F:GCATTCACACATATATTCTCAT  R:CAACTAACTCCAACTACAAAAA |
| InDel17 | *gyqtl10.1* | 153,270,143 | F:ATTGAGCGGAGATAAGAGAAC  R:GGAAGAAGGAATGATAAGATA |
| InDel20 | *gyqtl10.1* | 201,356,844 | F:TGAAGTGTCGTTTTCTGTT  R:GGTCTACTATTGTTGTGCG |

Table S3. Primer information for InDel labeling of the mature fruit Chr01 mapping interval

| Primer name | Positioning interval | Physical location | sequence（5'→3'） |
| --- | --- | --- | --- |
| InDel21 | *roqtl1.1* | 210,510,044 | F:ATGCCCAAGCAATCACTAAC  R:CACCAGGACCCAAACCAAAG |
| InDel23 | *roqtl1.1* | 221,738,426 | F:TACGAATAGCCAAAAGCAGAT  R:TCCCAAGGTAGGGGTAAGGTC |
| InDel25 | *roqtl1.1* | 231,736,123 | F:AGGTGATGGGTGTATCTCAGA  R:TGCCAGCCAAACTAGAAGTAT |
| InDel43 | *roqtl1.1* | 241,681,711 | F:TGGAATAAACATCACTCAAT  R:TTTCTACCTACACCACATAA |
| InDel29 | *roqtl1.1* | 253,483,242 | F:CAGAAGTAGATGAAGCCAAAT  R:GACAAAGTCACGAACGGTAAG |

Table S4. Primer information for InDel labeling of the mature fruit Chr06 mapping interval

| Primer name | Positioning interval | | Physical location | sequence（5'→3'） |
| --- | --- | --- | --- | --- |
| InDel32 | roqtl6.1 | | 2,878,137 | F:ATTTTGTTCTTATTCTTCGC  R:TATTTCAATCGGTCTCCCAC |
| InDel93 | roqtl6.1 | 5,904,977 | | F:CAAAATAACATAAGAGAAACAATAA  R:TAACTAAAATAAGGCCACGAAC |
| InDel129 | roqtl6.1 | 9,346,988 | | F:TACTTATTGTGTTTTCCTTTGA  R:ATTTATCTCTTGTAAACTCGTAAT |
| InDel136 | roqtl6.1 | 9,563,662 | | F:TCGGTGCTTGATATCTAGGG  R:GGAAAAAGGGTTTGGGGT |
| InDel137 | *roqtl6.1* | 9,803,501 | | F:ATCTTTTTCTGTTTTCGTTTTTAG  R:GATAACTCTGCCCACCAAAG |
| InDel57 | *roqtl6.1* | 9,901,304 | | F:GACTTGAGTAGATCCTGGA  R:ATGGGAAAGAGAAATAACA |
| InDel138 | *roqtl6.1* | 10,463,346 | | F:TCATCTGGTTTTCCCACTTC  R:TTCGTCTTTTCTTTTGCTCC |
| InDel122 | *roqtl6.1* | 10,829,149 | | F:TCACCTCCCATTAGCACAGA  R:GTTGTTGGAAGTGGAGAGTATG |
| InDel97 | *roqtl6.1* | 11,612,606 | | F:ACTGAAATTGGAGACATGAGTT  R:GCATTACAAACGAGATAGAACC |
| InDel130 | *roqtl6.1* | 12,062,527 | | F:GTCTTATAGTAAAATGGGTTGC  R:TTGTATTGTGTATGTATTAGGTGG |
| InDel35 | *roqtl6.1* | 12,553,933 | | F:TGTCTATGAACATGAGGTGC  R:AAAGTCAAGTTAATCCAACG |
| InDel99 | *roqtl6.1* | 12,667,654 | | F:AATGAGTCAAGGTGAAAGATG  R:GGAATGCCTAACTTGGTGA |
| InDel140 | *roqtl6.1* | 13,201,588 | | F:TCCCCCAACTAACTCCATCT  R:TATCACTATGACTTCAGTTACAATCTAT |
| InDel60 | *roqtl6.1* | 13,699,947 | | F:GCAATTTTCGTTTGGGTC  R:CGTCTTTCAAGTGGGGGA |
| InDel38 | *roqtl6.1* | 16,636,80 | | F:TTGTAAACAGACGAGAT  R:TCAAATGTATTTCAGCT |

Continued Table S4. Primer information for InDel labeling of the mature fruit Chr06 mapping interval

| Primer name | Positioning interval | Physical location | sequence（5'→3'） |
| --- | --- | --- | --- |
| InDel40 | *roqtl6.1* | 22,958,804 | F:CCGACCGATAGGTATGAGA  R:TTATTACTTTCCAGCGTTT |

Table S5. CAPCCS and CapGLK2 gene DNA cloning primer information

| Primer name | Sequence（5'→3'） |
| --- | --- |
| DNA-CCS-F | TTTCCATCTCCTTTACTT |
| DNA-CCS-R | GCTTTTGTTTCACTTTTG |
| GLK2-F | atggccatggaggccgaattcATGATGCTTGTTGTATCTACACCATTG |
| GLK2-R | ccgctgcaggtcgacggatccTCAAGTTGGAGGTATTTTTGTAATCC |

Table S6. *CAPCCS* gene QPCR primer information

| Primer name | Sequence（5'→3'） |
| --- | --- |
| qP-CCS-F | ACCCACATCAAAGCCAGAGTC |
| qP -CCS-R | TGAAGGGTCAACGCAACATAC |
| CA12g20490-F | GAAGACCCTGACGGGCAAGAC |
| CA12g20490-R | TTAGCACCACCACGGAGACGA |

Table S7. The procedures related to the green fruit color of the parent and the F_1_ generation

| generation | *L* | *C* | h | *ΔE* |
| --- | --- | --- | --- | --- |
| P_1_ | 61.87±1.77 a | 37.74±3.67 a | 101.50±3.18 a | 72.75±3.14 a |
| F_1_ | 43.89±1.58 b | 30.70±0.99 ab | 119.93±0.84 b | 53.56±1.84 b |
| P_2_ | 36.96±1.07 c | 23.53±1.22 b | 122.84±0.71 b | 44.36±1.56 c |

note：（Different lowercase letters indicate significant differences between different generations of green fruits (P <0.05)

Table S8. The correlation between the total chlorophyll content of the green fruit peel and the value of L, C, H, and E value

| index | *L v*alue | *C value* | *H* value | *ΔE* value |
| --- | --- | --- | --- | --- |
| Correlation coefficient | -0.851** | -0.921** | 0.839** | -0.918** |
| T statistics | 5.821 | 8.663 | 5.523 | 8.456 |
| T critical value(99%) | 2.821 | 2.821 | 2.821 | 2.821 |
| T critical value(95%) | 1.833 | 1.833 | 1.833 | 1.833 |

note：T statistical value> T critical value (99.9%) is represented by **, that is, the difference at 0.01 is significantly different

###

### Table S9. The procedure of the mature fruit color of the parent and the F_1_ generation

| generation | *L* | *C* | h | *ΔE* |
| --- | --- | --- | --- | --- |
| P_1_ | 56.52±1.09 a | 61.02±0.91 a | 70.21±1.22 a | 83.22±1.10 a |
| F_1_ | 37.11±0.84 b | 40.79±1.34 b | 30.69±0.96 b | 55.15±1.54 b |
| P_2_ | 34.85±0.92 b | 32.52±0.78 c | 26.57±0.97 b | 47.68±1.13 c |

note：Different lowercase letters indicate significant differences in different generations of green fruit color（P < 0.05）。

Table S10. The correlation between the total carotene content between the mature fruit peel and the value of the carotene content of L, C, H, and E

| index | *L v*alue | *C v*alue | *H v*alue | *ΔE v*alue |
| --- | --- | --- | --- | --- |
| Correlation coefficient | -0.907** | -0.900** | -0.924** | -0.915** |
| Tstatistics | 5.944 | 5.689 | 6.727 | 6.257 |
| Tcritical value(99%) | 3.143 | 3.143 | 3.143 | 3.143 |
| Tcritical value(95%) | 1.943 | 1.943 | 1.943 | 1.943 |

note：T statistical value> T critical value (99.9%) is represented by **, that is, significant differences at the level of 0.01.

Table S11.The genetic relationship between green fruit color and mature fruit color

| Mature  Fruitcolor | Green fruit color | | | | | Green  Fruit color | Mature fruit color | | | |
| --- | --- | --- | --- | --- | --- | --- | --- | --- | --- | --- |
|  | green | Yelllowish green | Pale green | *E* | *χ*^2^ |  | red | orange | *E* | *χ*^2^ |
| red | 268 | 86 | 23 | 12:3:1^*^ | 4.10 | green | 268 | 107 | 3:1^*^ | 2.49 |
| orange | 107 | 23 | 10 | 12:3:1^*^ | 0.62 | Yellowish green | 86 | 23 | 3:1^*^ | 0.88 |
| Total | 375 | 109 | 33 |  |  | Pale green | 23 | 10 | 3:1^*^ | 0.49 |
|  |  |  |  |  |  | total | 377 | 140 |  |  |

Note：E represents the separation ratio; 20.05, 1 = 3.84; 20.05, 2 = 5.99;*indicates that the difference at 0.05 is significantly different.

Table S12. Summary table of the whole genome sequencing data

| sample | Total number  of reads | Numberof base  (Gb) | Efficiene rate  (%) | Averae depth  (×) | Q20(%) | Q30(%) | GC content  (%) |
| --- | --- | --- | --- | --- | --- | --- | --- |
| D24 parental parent | 704,237,750 | 105.64 | 99.79% | 34.17 | 97 | 89.7 | 34.8 |
| D47 parental parent | 712,901,256 | 106.94 | 99.80% | 34.63 | 97.5 | 91.2 | 34.5 |
| Green immature fruit mixed pool | 1,064,991,646 | 159.75 | 99.74% | 51.45 | 96.8 | 89.4 | 34.9 |
| Yellowish immature  Fruit mixed pool | 1,119,049,564 | 167.86 | 99.63% | 54.34 | 97.5 | 91.3 | 34.2 |
| Mature red fruit mixed pool | 1,058,170,974 | 158.73 | 98.94% | 49.97 | 97.3 | 90.8 | 35.1 |
| Mature orange fruit mixed pool | 1,051,862,980 | 157.78 | 99.69% | 49.87 | 97.4 | 90.9 | 35.2 |

Table S13. Statistics of linkage interval information of green fruit color of pepper fruit

| TraitID | Chrom | Marker | Pos (bp) | LOD | PVE(%) | Add | Dom | CI |
| --- | --- | --- | --- | --- | --- | --- | --- | --- |
| *gyqtl1.1* | Chr01 | InDel141-InDel134 | 14,372,013-17,126,499 | 15.2865 | 19.6241 | 2.8014 | -0.5958 | 26.5-33.5 |
| *gyqtl10.1* | Chr10 | InDel107-InDel110 | 8,249,029-11,626,729 | 16.9089 | 22.0898 | 3.3691 | 0.7102 | 32.5-37.5 |

Table S14. Information statistics of the linkage interval of mature fruit color of pepper fruit

| TraitID | Chrom | Marker | Pos (bp) | LOD | PVE(%) | Add | Dom | CI |
| --- | --- | --- | --- | --- | --- | --- | --- | --- |
| *roqtl6.1* | Chr06 | InDel136-KASP | 9,563,662-9,717,156 | 115.0447 | 81.7015 | 20.7336 | -17.5957 | 23.5-24.5 |

Table S15. Function annotation of *gyqtl1.1* in Chr01 candidate

| Gene ID | Function annotation |
| --- | --- |
| Capann_59V1aChr01g007720.1 | putative ubiquitin-conjugating enzyme E2 25 [*Capsicum annuum*] |
| Capann_59V1aChr01g007730.1 | Ethylene-responsive transcription factor 5 [*Capsicum annuum*] |
| Capann_59V1aChr01g007740.1 | hypothetical protein FXO37_34830 [*Capsicum annuum*] |
| Capann_59V1aChr01g007750.1 | PREDICTED: ethylene-responsive transcription factor 1A-like [*Capsicum annuum*] |
| Capann_59V1aChr01g007760.1 | putative E3 ubiquitin-protein ligase LUL2 [*Capsicum annuum*] |
| Capann_59V1aChr01g007770.1 | PREDICTED: ethylene-responsive transcription factor 2-like [*Capsicum annuum*] |
| Capann_59V1aChr01g007780.1 | hypothetical protein CQW23_00798 [*Capsicum baccatum*] |
| Capann_59V1aChr01g007790.1 | pentatricopeptide repeat-containing protein At1g79490, mitochondrial [*Capsicum annuum*] |
| Capann_59V1aChr01g007800.1 | PREDICTED: ubiquitin carboxyl-terminal hydrolase 3 isoform X3 [*Capsicum annuum*] |
| Capann_59V1aChr01g007810.1 | PREDICTED: probable enoyl-CoA hydratase 2, mitochondrial isoform X2 [*Capsicum annuum*] |
| Capann_59V1aChr01g007820.1 | putative pentatricopeptide repeat-containing protein, mitochondrial-like [*Capsicum annuum*] |
| Capann_59V1aChr01g007830.1 | PREDICTED: probable polyamine transporter At1g31830 isoform X1 [*Capsicum annuum*] |
| Capann_59V1aChr01g007840.1 | Phospholipase A1-Ibeta2, chloroplastic [*Capsicum annuum*] |
| Capann_59V1aChr01g007850.1 | Pentatricopeptide repeat-containing protein, mitochondrial [*Capsicum annuum*] |
| Capann_59V1aChr01g007860.1 | Pentatricopeptide repeat-containing protein, mitochondrial [*Capsicum annuum*] |
| Capann_59V1aChr01g007870.1 | hypothetical protein CQW23_00788 [*Capsicum baccatum*] |
| Capann_59V1aChr01g007880.1 | PREDICTED: uncharacterized protein LOC107868293 [*Capsicum annuum*] |

Continued Table S15. Function annotation of *gyqtl1.1* in Chr01 candidate

| Gene ID | Function annotation |
| --- | --- |
| Capann_59V1aChr01g007890.1 | PREDICTED: FAD-dependent urate hydroxylase-like [*Capsicum annuum*] |
| Capann_59V1aChr01g007900.1 | PREDICTED: uncharacterized protein LOC107853126 isoform X1 [*Capsicum annuum*] |
| Capann_59V1aChr01g007910.1 | PREDICTED: proline-rich receptor-like protein kinase PERK2 [*Capsicum annuum*] |
| Capann_59V1aChr01g007920.1 | PREDICTED: 50S ribosomal protein L19-2, chloroplastic-like [*Capsicum annuum*] |
| Capann_59V1aChr01g007930.1 | PREDICTED: vesicle-associated protein 2-1-like [*Capsicum annuum*] |
| Capann_59V1aChr01g007940.1 | PREDICTED: uncharacterized protein LOC107868332 [*Capsicum annuum*] |
| Capann_59V1aChr01g007950.1 | Bax inhibitor 1 [*Capsicum annuum*] |
| Capann_59V1aChr01g007960.1 | PREDICTED: uncharacterized protein LOC107868359 [*Capsicum annuum*] |
| Capann_59V1aChr01g007970.1 | PREDICTED: putative CCA tRNA nucleotidyltransferase 2 isoform X1 [*Capsicum annuum*] |
| Capann_59V1aChr01g007980.1 | GATA transcription factor 26 [*Capsicum annuum*] |
| Capann_59V1aChr01g007990.1 | hypothetical protein FXO37_24181 [*Capsicum annuum*] |
| Capann_59V1aChr01g008000.1 | Cytochrome 94A2 [*Capsicum baccatum*] |
| Capann_59V1aChr01g008010.1 | PREDICTED: uncharacterized protein LOC107868389 [*Capsicum annuum*] |
| Capann_59V1aChr01g008020.1 | putative GATA transcription factor 26-like [*Capsicum annuum*] |
| Capann_59V1aChr01g008030.1 | PREDICTED: uncharacterized protein LOC107868408 [*Capsicum annuum*] |
| Capann_59V1aChr01g008040.1 | hypothetical protein FXO38_08719 [*Capsicum annuum*] |
| Capann_59V1aChr01g008040.2 | hypothetical protein FXO38_08719 [*Capsicum annuum*] |

Continued Table S15. Function annotation of *gyqtl1.1* in Chr01 candidate

| Gene ID | Function annotation |
| --- | --- |
| Capann_59V1aChr01g008050.1 | hypothetical protein FXO38_08718 [*Capsicum annuum*] |
| Capann_59V1aChr01g008060.1 | hypothetical protein CQW23_00773 [*Capsicum baccatum*] |
| Capann_59V1aChr01g008070.1 | PREDICTED: expansin-like B1 [*Capsicum annuum*] |
| Capann_59V1aChr01g008080.1 | Expansin-like B1 [*Capsicum annuum*] |
| Capann_59V1aChr01g008090.1 | PREDICTED: uncharacterized protein LOC107841403 [*Capsicum annuum*] |
| Capann_59V1aChr01g008100.1 | hypothetical protein FXO38_01282 [*Capsicum annuum*] |
| Capann_59V1aChr01g008110.1 | ATP synthase F1 subunit 1 [*Capsicum annuum*] |
| Capann_59V1aChr01g008120.1 | hypothetical protein FXO37_28467 [*Capsicum annuum*] |
| Capann_59V1aChr01g008130.1 | hypothetical protein FXO38_33925 [*Capsicum annuum*] |
| Capann_59V1aChr01g008140.1 | NADH-ubiquinone oxidoreductase chain 2 [*Cucumis melo* var. *makuwa*] |
| Capann_59V1aChr01g008150.1 | hypothetical protein T459_33116 [*Capsicum annuum*] |
| Capann_59V1aChr01g008160.1 | NADH dehydrogenase subunit [*Dorcoceras hygrometricum*] |
| Capann_59V1aChr01g008170.1 | hypothetical protein FXO38_01282 [*Capsicum annuum*] |
| Capann_59V1aChr01g008180.1 | putative ATPase, F1 complex, alpha subunit, P-loop containing nucleoside triphosphate hydrolase [*Helianthus annuus*] |
| Capann_59V1aChr01g008190.1 | hypothetical protein [*Capsicum annuum*] |
| Capann_59V1aChr01g008200.1 | hypothetical protein FXO37_28467 [*Capsicum annuum*] |
| Capann_59V1aChr01g008210.1 | NADH-ubiquinone oxidoreductase chain 2 [*Cucumis melo* var. *makuwa*] |

Continued Table S15. Function annotation of *gyqtl1.1* in Chr01 candidate

| Gene ID | Function annotation |
| --- | --- |
| Capann_59V1aChr01g008220.1 | hypothetical protein T459_33116 [*Capsicum annuum*] |
| Capann_59V1aChr01g008230.1 | hypothetical protein FXO37_17106 [*Capsicum annuum*] |
| Capann_59V1aChr01g008240.1 | hypothetical protein [*Tanacetum cinerariifolium*] |
| Capann_59V1aChr01g008250.1 | hypothetical protein T459_28137 [*Capsicum annuum*] |
| Capann_59V1aChr01g008260.1 | hypothetical protein NitaMp081 [*Nicotiana tabacum*] |
| Capann_59V1aChr01g008270.1 | nadh-ubiquinone oxidoreductase chain 5 [*Nicotiana attenuata*] |
| Capann_59V1aChr01g008280.1 | hypothetical protein FXO37_07920 [*Capsicum annuum*] |
| Capann_59V1aChr01g008290.1 | hypothetical protein FXO38_34125 [*Capsicum annuum*] |
| Capann_59V1aChr01g008300.1 | ribosomal protein S12 [*Capsicum annuum*] |
| Capann_59V1aChr01g008310.1 | ATPase subunit 4, partial [*Trifolium pratense*] |
| Capann_59V1aChr01g008320.1 | NADH-ubiquinone oxidoreductase chain 1 [*Capsicum annuum*] |
| Capann_59V1aChr01g008330.1 | hypothetical protein C4D60_Mb00t12500 [*Musa balbisiana*] |
| Capann_59V1aChr01g008340.1 | Cell differentiation protein RCD1 -like protein [*Capsicum baccatum*] |
| Capann_59V1aChr01g008350.1 | hypothetical protein [*Capsicum annuum*] |
| Capann_59V1aChr01g008360.1 | hypothetical protein FXO38_27189 [*Capsicum annuum*] |
| Capann_59V1aChr01g008370.1 | PREDICTED: expansin-like B1 [*Capsicum annuum*] |
| Capann_59V1aChr01g008380.1 | PREDICTED: expansin-like B1 [*Capsicum annuum*] |

Continued Table S15. Function annotation of *gyqtl1.1* in Chr01 candidate

| Gene ID | Function annotation |
| --- | --- |
| Capann_59V1aChr01g008390.1 | PREDICTED: expansin-like B1 [*Capsicum annuum*] |
| Capann_59V1aChr01g008400.1 | ATP-dependent Clp protease proteolytic subunit-related protein 4, chloroplastic [*Capsicum annuum*] |
| Capann_59V1aChr01g008410.1 | Ankyrin repeat family protein [*Capsicum annuum*] |
| Capann_59V1aChr01g008420.1 | PREDICTED: ankyrin repeat-containing protein At3g12360-like [*Capsicum annuum*] |
| Capann_59V1aChr01g008430.1 | hypothetical protein FXO37_07601 [*Capsicum annuum*] |
| Capann_59V1aChr01g008440.1 | PREDICTED: uncharacterized protein LOC107868485 [*Capsicum annuum*] |
| Capann_59V1aChr01g008450.1 | PREDICTED: uncharacterized protein LOC107868493 isoform X2 [*Capsicum annuum*] |
| Capann_59V1aChr01g008460.1 | PREDICTED: uncharacterized protein LOC107868516 isoform X1 [*Capsicum annuum*] |
| Capann_59V1aChr01g008470.1 | PREDICTED: (S)-ureidoglycine aminohydrolase [*Capsicum annuum*] |
| Capann_59V1aChr01g008480.1 | Deoxyhypusine hydroxylase [*Capsicum annuum*] |
| Capann_59V1aChr01g008490.1 | PREDICTED: uncharacterized protein LOC107868570 [*Capsicum annuum*] |
| Capann_59V1aChr01g008500.1 | hypothetical protein CQW23_19082 [*Capsicum baccatum*] |
| Capann_59V1aChr01g008520.1 | putative cdc6 [*Capsicum annuum*] |
| Capann_59V1aChr01g008530.1 | PREDICTED: serine/threonine-protein kinase SAPK3-like [*Capsicum annuum*] |
| Capann_59V1aChr01g008540.1 | putative HUA2-like protein 3-like [*Capsicum annuum*] |
| Capann_59V1aChr01g008550.1 | hypothetical protein FXO38_11182 [*Capsicum annuum*] |
| Capann_59V1aChr01g008560.1 | PREDICTED: calcineurin B-like protein 1 [*Capsicum annuum*] |

Continued Table S15. Function annotation of *gyqtl1.1* in Chr01 candidate

| Gene ID | Function annotation |
| --- | --- |
| Capann_59V1aChr01g008570.1 | Pectin acetylesterase 7, partial [*Capsicum annuum*] |
| Capann_59V1aChr01g008580.1 | putative LOB domain-containing protein 24-like [*Capsicum annuum*] |
| Capann_59V1aChr01g008590.1 | hypothetical protein CQW23_00748 [*Capsicum baccatum*] |
| Capann_59V1aChr01g008600.1 | putative nephrocystin-3-like [*Capsicum annuum*] |
| Capann_59V1aChr01g008610.1 | PREDICTED: decapping nuclease DXO homolog, chloroplastic isoform X1 [*Capsicum annuum*] |
| Capann_59V1aChr01g008620.1 | putative eukaryotic translation initiation factor 4G-like [*Capsicum annuum*] |
| Capann_59V1aChr01g008630.1 | PREDICTED: receptor-like protein 12 [*Capsicum annuum*] |
| Capann_59V1aChr01g008640.1 | PREDICTED: receptor-like protein 12 [*Capsicum annuum*] |
| Capann_59V1aChr01g008650.1 | hypothetical protein FXO38_07740 [*Capsicum annuum*] |
| Capann_59V1aChr01g008660.1 | putative 60S ribosomal protein L37a-like [*Capsicum annuum*] |
| Capann_59V1aChr01g008670.1 | 60S ribosomal protein L37a [*Solanum tuberosum*] |
| Capann_59V1aChr01g008680.1 | putative receptor-like protein 12-like [*Capsicum annuum*] |
| Capann_59V1aChr01g008690.1 | hypothetical protein CQW23_00741 [*Capsicum baccatum*] |
| Capann_59V1aChr01g008700.1 | putative chromodomain-helicase-DNA-binding protein 8-like [*Capsicum annuum*] |
| Capann_59V1aChr01g008710.1 | hypothetical protein FXO37_31621 [*Capsicum annuum*] |
| Capann_59V1aChr01g008720.1 | putative omega-hydroxypalmitate O-feruloyl transferase-like [*Capsicum annuum*] |
| Capann_59V1aChr01g008740.1 | putative transcriptional regulator ATRX -like protein [*Capsicum annuum*] |

Continued Table S15. Function annotation of *gyqtl1.1* in Chr01 candidate

| Gene ID | Function annotation |
| --- | --- |
| Capann_59V1aChr01g008750.1 | PREDICTED: UMP-CMP kinase 3-like [*Capsicum annuum*] |
| Capann_59V1aChr01g008760.1 | PREDICTED: SUPPRESSOR OF ABI3-5 isoform X1 [*Capsicum annuum*] |
| Capann_59V1aChr01g008770.1 | PREDICTED: pentatricopeptide repeat-containing protein At3g29230-like [*Capsicum annuum*] |
| Capann_59V1aChr01g008780.1 | PREDICTED: expansin-like B1 isoform X1 [*Capsicum annuum*] |
| Capann_59V1aChr01g008790.1 | haloacid dehalogenase-like hydrolase domain-containing protein Sgpp [*Capsicum annuum*] |
| Capann_59V1aChr01g008800.1 | Dynamin-related protein 1E [*Capsicum annuum*] |
| Capann_59V1aChr01g008810.1 | PREDICTED: probable purine permease 11 [*Capsicum annuum*] |
| Capann_59V1aChr01g008820.1 | hypothetical protein FXO37_25390 [*Capsicum annuum*] |
| Capann_59V1aChr01g008830.1 | hypothetical protein FXO37_05838 [*Capsicum annuum*] |
| Capann_59V1aChr01g008840.1 | hypothetical protein T459_00768 [*Capsicum annuum*] |
| Capann_59V1aChr01g008850.1 | Histone-lysine N-methyltransferase ASHR3 [*Capsicum chinense*] |
| Capann_59V1aChr01g008860.1 | hypothetical protein BC332_00706 [*Capsicum chinense*] |

Table S16. Function annotation of *gyqtl1.1* in Chr10 candidate

| Gene ID | Function annotation |
| --- | --- |
| Capann_59V1aChr10g003440.1 | Acyl-coenzyme A oxidase 3, peroxisomal, partial [*Capsicum annuum*] |
| Capann_59V1aChr10g003450.1 | Acyl-coenzyme A oxidase 3, peroxisomal [*Capsicum annuum*] |
| Capann_59V1aChr10g003460.1 | putative acyl-coenzyme A oxidase 3, peroxisomal-like isoform X2 [*Capsicum annuum*] |
| Capann_59V1aChr10g003470.1 | PREDICTED: trans-resveratrol di-O-methyltransferase-like [*Capsicum annuum*] |
| Capann_59V1aChr10g003480.1 | PREDICTED: agamous-like MADS-box protein AGL86 [*Capsicum annuum*] |
| Capann_59V1aChr10g003490.1 | hypothetical protein T459_01541 [*Capsicum annuum*] |
| Capann_59V1aChr10g003500.1 | Prohibitin-6, mitochondrial [*Capsicum annuum*] |
| Capann_59V1aChr10g003510.1 | hypothetical protein FXO37_15460 [*Capsicum annuum*] |
| Capann_59V1aChr10g003520.1 | hypothetical protein FXO38_07989 [*Capsicum annuum*] |
| Capann_59V1aChr10g003530.1 | Glucosidase 2 subunit beta [*Capsicum baccatum*] |
| Capann_59V1aChr10g003540.1 | putative dolichyl-diphosphooligosaccharide--protein glycosyltransferase subunit 3 [*Capsicum baccatum*] |
| Capann_59V1aChr10g003550.1 | PREDICTED: elongation factor P-like [*Capsicum annuum*] |

Table S17. Key candidate genes in the QTL linkage interval of green fruit color

| Gene name | Physical location(bp) | Note name | QTL |
| --- | --- | --- | --- |
| Capann_59V1aChr10g003610 | 9,177,688-9,185,257 | *GLK2* | *gyqtl10.1* |

Table S18. Function annotation of the *roqtl6.1* gene between Chor06 candidate

| Gene ID | Function annotation |
| --- | --- |
| Capann_59V1aChr06g006040.1 | PREDICTED: protein SELF-PRUNING [*Capsicum annuum*] |
| Capann_59V1aChr06g006050.1 | PREDICTED: zeatin O-glucosyltransferase-like [*Capsicum annuum*] |
| Capann_59V1aChr06g006060.1 | PREDICTED: zeatin O-glucosyltransferase-like [*Capsicum annuum*] |
| Capann_59V1aChr06g006070.1 | Uridine 5'-monophosphate synthase [*Capsicum annuum*] |
| Capann_59V1aChr06g006080.1 | PREDICTED: uncharacterized protein LOC107875653 [*Capsicum annuum*] |
| Capann_59V1aChr06g006090.1 | Transcription factor HBP-1b(c1) [*Capsicum annuum*] |
| Capann_59V1aChr06g006100.1 | putative sphinganine C(4)-monooxygenase 1-like [*Capsicum annuum*] |

Continued Table S18. Function annotation of the *roqtl6.1* gene between Chor06 candidate

| Gene ID | Function annotation |
| --- | --- |
| Capann_59V1aChr06g006120.1 | PREDICTED: 60S ribosomal protein L10a-1 [*Capsicum annuum*] |
| Capann_59V1aChr06g006130.1 | 60S ribosomal protein L10a [*Capsicum annuum*] |
| Capann_59V1aChr06g006140.1 | putative protein FAF-like, chloroplastic-like [*Capsicum annuum*] |
| Capann_59V1aChr06g006150.1 | PREDICTED: protein FAF-like, chloroplastic [*Capsicum annuum*] |
| Capann_59V1aChr06g006170.1 | PREDICTED: uncharacterized protein LOC107875662 [*Capsicum annuum*] |
| Capann_59V1aChr06g006180.1 | Vesicle-associated membrane protein [*Capsicum annuum*] |
| Capann_59V1aChr06g006190.1 | Vesicle-associated membrane protein [*Capsicum annuum*] |
| Capann_59V1aChr06g006200.1 | RecName: Full=Capsanthin/capsorubin synthase, chromoplastic; Flags: Precursor [*Capsicum annuum*] |

Table S19. Key candidate genes in the QTL linkage interval of old ripe fruit color

| Gene name | Physical location（bp） | Note name | QTL |
| --- | --- | --- | --- |
| Capann_59V1aChr06g006200 | 9,715,949-9,717,388 | *CCS* | *roqtl6.1* |
